# A voting approach to identify a small number of highly predictive genes using multiple classifiers

**DOI:** 10.1186/1471-2105-10-S1-S19

**Published:** 2009-01-30

**Authors:** Md Rafiul Hassan, M Maruf Hossain, James Bailey, Geoff Macintyre, Joshua WK Ho, Kotagiri Ramamohanarao

**Affiliations:** 1 Department of Computer Science and Software Engineering, The University of Melbourne, Victoria 3010, Australia; 2 NICTA Victoria Laboratory, The University of Melbourne, Victoria 3010, Australia; 3 School of Information Technologies, The University of Sydney, NSW 2006, Australia; 4 NICTA, Australian Technology Park, Eveleigh, NSW 2015, Australia

## Abstract

**Background:**

Microarray gene expression profiling has provided extensive datasets that can describe characteristics of cancer patients. An important challenge for this type of data is the discovery of gene sets which can be used as the basis of developing a clinical predictor for cancer. It is desirable that such gene sets be compact, give accurate predictions across many classifiers, be biologically relevant and have good biological process coverage.

**Results:**

By using a new type of multiple classifier voting approach, we have identified gene sets that can predict breast cancer prognosis accurately, for a range of classification algorithms. Unlike a wrapper approach, our method is not specialised towards a single classification technique. Experimental analysis demonstrates higher prediction accuracies for our sets of genes compared to previous work in the area. Moreover, our sets of genes are generally more compact than those previously proposed. Taking a biological viewpoint, from the literature, most of the genes in our sets are known to be strongly related to cancer.

**Conclusion:**

We show that it is possible to obtain superior classification accuracy with our approach and obtain a compact gene set that is also biologically relevant and has good coverage of different biological processes.

## Background

Gene microarrays are a popular technology for assisting with the prediction and understanding of diseases [1,2]. Cancer is one such disease where this technology has proved to be particularly powerful. An important challenge in this area is the discovery of gene sets which can be used as predictors of cancer. For a gene set to be useful as the basis of developing a clinical predictor for cancer, there are a number of desirable properties it should have:

• *Compactness*: There should not be too many genes in the set. This reduces the cost involved in developing a clinical diagnostic test using these genes.

• *Accuracy*: When the genes are input to a machine learning algorithm as features, it should be possible to achieve a high true positive rate and a low false positive rate.

• *Classifier independence*: It should be possible to achieve high accuracy using a range of different machine learning classifiers with the gene set. This increases the confidence that biologists have in the stability and generality of the gene set.

• *Biological relevance*: Most of the genes in the gene set should have a known relationship to cancer, based on the literature.

• *Biological coverage*: The genes in the gene set should span a number of distinct biological processes and each gene should be independently useful for prediction. The set of genes should not be confined to a single pathway. This increases the robustness of prediction and allows more uniform classification power across different subtypes of cancer.

In this paper, we propose a new classifier voting approach to discover a gene set for breast cancer prognosis that satisfies these five properties. We are able to discover a gene set using the van 't Veer *et al. *[3] dataset that consists of 7 genes and delivers highly accurate prediction results for a range of classifiers. In addition, we were able to discover a 6 gene set that delivers high accuracy on the Ma *et al. *[4] dataset, for which we also validated our performance on an additional independent dataset exhibiting the same biological conditions [5]. The majority of these genes have been previously mentioned in the cancer literature, and we have found the genes in these sets to be relatively independent in terms of function, meaning our genes are able to cover a number of different processes involved in cancer. In comparison with other studies on these datasets, our gene sets are considerably smaller and deliver considerably higher performance across a range of machine learning classifiers.

Our proposed technique is based on the use of multiple voting classifiers to identify the final gene set. Its use of multiple classifiers makes it different from previous work for microarray classification, such as wrapper based methods, which only target a single classifier.

An important aspect of our method is that it does not employ any biological domain knowledge (e.g. the Gene Ontology) as part of the algorithm for identifying the gene set. This makes it particularly applicable for deployment in scenarios where the literature is sparse or the state-of-the-art is immature. Nevertheless, for the dataset we use, we are able to confirm that the individual genes in the sets that are discovered are biologically relevant.

## Methods

Our proposed approach comprises two steps. In the first step, we rank all genes of the training set. In the second step, we investigate the classification performance of combinations of genes using a voting approach from the ranked genes obtained from the first step, but employing a number of classifiers instead of just one classifier. The steps are described in detail in the subsequent sections.

### The receiver operating characteristic (ROC) curve: preliminaries

In machine learning, the receiver operating characteristic (ROC) curve is used to evaluate the discriminative performance of binary classifiers. This is obtained by plotting the curve of the true positive rate (*Sensitivity*) versus the false positive rate (1 – *Specificity*) for a binary classifier by varying the discrimination threshold.

All the calculations of true positive rate and false positive rate are attained when using a particular classifier threshold. By varying the threshold, a set of values for these measurements is obtained. This set of values is plotted in a two-dimensional Cartesian graph to yield the ROC curve. The ROC curve takes into account all the possible solutions by varying the discriminative threshold. The best performance would be produced, if the ROC curve matches with the upper left corner of the ROC space (which yields 100% *sensitivity *and 100% *specificity*). The closer the ROC curve is to the upper part of the ROC space, the better the performance of the classifier.

An ROC curve is a two dimensional illustration of classifier performance. Reducing ROC performance to a single scalar value to represent expected performance helps compare classifiers. A popular method is to calculate the area under the ROC curve (AUC) [6].

The AUC, being a part of the area of the unit square, has a value between 0 and 1. Since random guessing could produce the diagonal line between (0, 0) and (1, 1) with an area of 0.5, a classifier with an AUC less than 0.5 is undesirable [7]. An AUC value close to 1 indicates better performance for a binary classifier [8].

### Feature ranking using ROC

We rank all genes of the training set using the first step of Mamitsuka [9]'s ROC, which is the equivalent of the Mann-Whitney U statistic [10], normalized by the number of possible pairings of positive and negative values, also known as the two sample Wilcoxon rank sum statistic [6]. The AUC actually represents the probability that a randomly chosen positive example is correctly rated (ranked) with greater suspicion than a randomly chosen negative example.

Let us consider a training dataset D of *n *examples, where each example comprises *m *attributes: *x*_1_, *x*_2_, *x*_3_,..., *x*_*m*_. Each of the *m *attributes has a differing discriminative power reflected by its respective AUC. To calculate the discriminative power that is expressed in terms of AUC, we plot the ROC curve for each gene paired with the class label, (i.e., {*x*_*i*_, *Y*_*i*_}, where 1 ≤ *i *≤ *m *and *Y *is the vector of class labels) and calculate the AUC of the ROC curve. Now, we order the genes based on their respective AUCs.

### Multi-classifier voting approach to select genes

We attempt to classify the validation dataset with the top ranked genes. At first we pass the top 10 genes individually to all classifiers, and note the classification accuracy of each classifier on a validation set. For the second pass, we select the gene for which the most classifiers achieve their highest accuracy. Then we form a pair of the selected gene from the first pass, along with the remaining nine genes, and input these nine pairs to the selected classifiers. We then note the classification accuracy. The pair on which the most classifiers achieve their highest accuracy is selected and given to a third pass. We continue adding single genes to form 3-gene combinations, and so on.

A diverse set of fifteen classifiers was used for this process. They are: Logistic Model Tree (LMT) [11], Naïve Bayes Tree (NBTree) [12], Naïve Bayes, Random Forest, C4.5, *k*-Nearest Neighbour (*k*-NN), Artificial Neural Network (ANN), Logistic Regression, Support Vector Machine (SVM), and bagging [13] and boosting (ADABoost.M1) [14] for Naïve Bayes, Random Forest and C4.5.

We stop growing the gene set once more than 50% of the classifiers have their accuracy lowered on the validation set by the addition of an individual gene. In the case of any tie between two or more genes, we note the total accuracy (out of 1500%) for the tied genes and the gene with the largest total accuracy is chosen for the next pass. This majority voting approach allows us to select a small subset of genes that can boost classification accuracy on a number of classifiers. One can then tune the performance of an individual classifier by choosing the prefix of the genes (ranked using the voting approach) that delivers best accuracy. For example, for C4.5 with boosting on the van 't Veer data, adding the 5th gene to the first four genes actually degrades the performance of that specific classifier. So for that individual classifier, rather than using our final selection of 7 genes, we can instead use only a subset of 4 (out of the 7) genes.

### Datasets

In each of the three datasets used in our analysis, the prognostic outcome to be predicted is whether distant metastases will occur within 5 years (poor prognosis) or whether the patient is disease-free after 5 years (good prognosis).

*van't Veer data *[3]: The dataset comprises 97 breast cancer patients treated through modified radical mastectomy or breast-conserving treatment followed by radiotherapy. The patients were split into a training set of 68, a validation set of 10, and a test set of 19 cases. The training set consists of 29 positive (poor prognoses) and 39 negative (good prognoses) cases, the validation set comprises five positive and five negative cases, and the test set was made up of 12 positive and 7 negative cases. Further, we created a merged dataset from van 't Veer's [3] training (our training and validation set) and test sets to apply *k*-fold cross validation (CV). In *k*-fold CV, 10 cases out of the training set are randomly selected for the validation set before applying the FROC.

*Ma et al. data *[4]: This dataset contains 60 breast cancer patients treated through standard breast surgery followed by continued adjuvent tamoxifen therapy. There were 28 positive cases (poor prognoses) and 32 negative cases (good prognoses). We separate the first 5 positive cases and the last 5 negative cases to form the test set and use the remaining cases for training.

*Loi et al. data *[5]: The dataset is made up of 77 breast cancer patients obtained from the GUYT2 test data used in the Loi *et al*. study, with similar treatments to those performed in the Ma *et al. *dataset. There were 10 positive cases (poor prognoses) and 67 negative cases (good prognoses). This dataset was included in our study as a completely independent test dataset. All patients were considered as test cases to gauge performance of the classifiers trained on the Ma *et al. *dataset.

### Evaluation

For the van 't Veer [3] and Ma *et al. *[4] datasets, we used a holdout cross validation procedure, where a training set is used to train the classifiers and a separate test set is used to evaluate. We have also used the more general *k*-fold cross validation (CV) scheme to evaluate the performance on van 't Veer data. In *k*-fold CV, the original sample is partitioned into *k *subsamples. Of the *k *subsamples, a single subsample is retained as the validation data for testing the model, and the remaining *k *- 1 subsamples are used as training data. The CV process is then repeated *k *times (the folds), with each of the *k *subsamples used exactly once as the validation data. The *k *results from the folds, then, can be averaged (or otherwise combined) to produce a single estimation. Here, we have chosen *k *to be 5. The results for *k*-fold CV is presented in the Additional File 1.

All of our evaluation results are reported in weighted accuracy [15], which is calculated by the formula shown in Eq. (1).

(1) $$\text{Weighted Accuracy}=\frac{\frac{TP}{P}+\frac{TN}{N}}{2}$$

where, *P *= Total number of positive cases,

*N *= Total number of negative cases.

## Results

Using the van 't Veer data [3], our final selected gene set consists of the 7 genes (*TSPYL5*, *NMU*, *CA9*, *AGTPBP1*, *LIN9*, *ASPM*, and *DIAPH1*) and as we shall show, this compact gene set delivers highly accurate performance across a range of classifiers. The functions of these genes are summarised in Table 1. Additionally, we used the Ma *et al. *[4] data to test whether our method was successful on a dataset with different biology to the van 't Veer dataset. We have obtained a different set of 6 genes (*RGS19*, *ZIC2*, *SRD5A3*, *PPARD*, *GM2A*, *CD55*). We also tested the generalisability of this 6 gene set on an additional independent dataset exhibiting the same biological conditions [5]. Most of the later discussion will be using the van 't Veer data as an example, unless otherwise stated.

**Table 1:** Our set of 7 genes selected by majority voting and ordered by area under ROC curve

**GeneBank Accession Number**	**AUC**	**Gene Symbol**	**Gene Description**
AL080059	0.800802	*TSPYL5*	TSPY-like 5

NM_006681	0.794786	*NMU*	Neuromedin U

NM_001216	0.794786	*CA9*	Carbonic Anhydrase IX

AA830802	0.792781	*AGTPBP1*	ATP/GTP binding protein 1

AA834945	0.774733	*LIN9*	Lin-9 homolog (C. elegans)

AA748494	0.766711	*ASPM*	ASP (abnormal spindle) homolog, microcephaly associated (Drosophila)

NM_005219	0.764706	*DIAPH1*	Diaphanous homolog 1 (Drosophila)

### Biological significance of the compact gene sets

As the treatment procedures applied to the patients in both the van 't Veer study [3] (no adjuvent therapy) and Ma *et al. *study [4] (adjuvent therapy with tamoxifen) are vastly different, it is not surprising that there is no overlap between the two gene sets identified as the best predictors of prognosis outcome. The biology driving the chance to distant metastasis in each dataset is likely to be significantly different and as such it would not make sense to expect the gene lists to overlap. Therefore, we will consider each gene set independently.

Gene Ontology (GO) and Kyoto Encyclopedia of Genes and Genomes (KEGG) pathway analysis of the two gene sets show that each of the genes are diverse in function and appear to be unrelated in terms of the biological processes in which they are involved. What is interesting, however, is that the majority of the genes have been previously shown to be related to cancer in the literature (as shown below). This suggests that our feature selection procedure yields a compact sampling of the diverse biological processes represented by the microarray, which are highly representative of the prognostic potential of the patient. In concordance with this, in the 7 gene set, *TSPYL5 *and *CA9 *have been previously used as prognostic biomarkers in cancer [15-19]. Furthermore, four of the top 7 genes selected by our method are in the set of 231 genes used in the study by van 't Veer [3]. and the most important individual gene in improving a number of the classifier performances in the test set (*TSPYL5*) is present in the 17 genes selected by Alexe *et al. *[15]. In the 6 gene set, *CD55 *has been used previously as a prognostic biomarker in gastric cancer [20].

### Links between identified genes and the cancer literature

#### 7 gene set

Each of the 7 genes can be directly linked to potential cancer re-occurrence through their respective biological functions. *TSPYL5 *is involved in nucleosome assembly, a process which, if destabilised, can alter the regulatory mechanisms of a cell [21], which is likely to occur in cancer. *NMU *has been shown to be related to metastatic potential and cancer cachexia [22], which would have a significant impact on the potential of reoccurrence of the cancer. *CA9 *is involved in nitrogen metabolism and is linked to cell proliferation and *ASPM *is involved in mitotic spindle regulation and is expressed in proliferating tissues. (Proliferation is a mechanism which is well known to be related to the cancerous potential of cells). *LIN9 *is involved in progression through the cell cycle [23] and is a tumor suppressor [24] that inhibits DNA synthesis, thus having significant cancerous potential. Regulation of the *DIAPH1 *gene [25] is important in regulating the transcription factor Mitf, which in turn regulates the invasiveness of melanoma. Finally, while no significant link to cancer processes were found for *AGTPBP1*, somatic mutations in the coding sequence have been found in colorectal cancers [26].

#### 6 gene set

5 out of the 6 genes in this set not only have links to cancer through the literature, they have in most cases been shown to be directly linked to prognostic outcome. A study into antibodies against *ZIC2 *in small lung cell carcinoma showed that the concentration of antibodies is a good indicator of prognosis [27]. *SRDA53 *overexpression in hormone-refractory prostate cancers was shown to be crucial for cell viability [28] and is a likely factor in resistance to hormone based therapies in prostate cancers. *PPAR *has been shown previously to be abberently expressed in colon cancer cells [29] and is an important player in the proliferation and growth of these cells [30]. A protein involved in innate immune response which is critical to the regulation of the complement cascade, *CD55*, has been shown to be important in prostate growth [31], gastric tumor invasiveness [32] and breast cancer prognosis [33]. Finally, *RGS19 *has been implicated in the control of autophagy in colon cancer cell lines [34].

The demonstrated links of these genes with the literature highlights the relevancy of each of the genes with respect to cancer and demonstrates their potential to represent biological processes which are directly related to the prognostic potential (chance of cancer re-occurrence) of a patient.

### Classification performance on test set

#### 7 gene set

Table 2 shows the results for a range of different classifiers being tested on the van 't Veer [3] dataset, when configured to select their preferred subset of genes from our 7 gene set. As most classifiers have their own internal mechanism to rank and select the features to classify, it is obvious that all classifiers will not perform similarly with the same subset of genes. The 'majority voting' scheme, used to select the significant 7 genes in our multi-classifier voting approach from one pass to another, helped in improving the performance of the all classifiers. However, a few classifiers – namely C4.5, C4.5 with bagging, Naïve Bayes with bagging, Naïve Bayes with boosting, LMT, NBTree and *k*-NN – showed the best performance using only a single gene.

**Table 2:** Accuracy achieved on test set by different classifiers using various subsets of our 7 genes

**Classifier**	**Accuracy**	**Gene Combination**
C4.5	84.52%	*TSPYL5*

C4.5 with boosting (ADABoost.M1)	91.67%	*TSPYL5-DIAPH1-AGTPBP1*

C4.5 with bagging	84.52%	*TSPYL5*

Naïve Bayes	84.52%	*TSPYL5*

Naïve Bayes with boosting	84.52%	*TSPYL5*

Naïve Bayes with bagging	88.69%	*TSPYL5-DIAPH1-NMU*

LMT	84.52%	*TSPYL5*

NBTree	84.52%	*TSPYL5*

Random Forest	84.52%	*TSPYL5-DIAPH1-ASPM*

Random Forest with boosting	84.52%	*TSPYL5-DIAPH1-ASPM*

Random Forest with bagging	88.69%	*TSPYL5-DIAPH1-ASPM-NMU*

*k*-NN	80.36%	*TSPYL5*

Logistic Regression	81.55%	*TSPYL5-DIAPH1-CA9*

ANN	77.38%	*TSPYL5-CA9*

SVM	83.33%	*TSPYL5-LIN9*

The best performance for the test dataset obtained was 91.67% for C4.5 with boosting. This performance was achieved using only three genes: *TSPYL5, DIAPH1*, and *AGTPBP1*. It is worth noting that the gene subset {*TSPYL5*, *DIAPH1*} was found to be significant for at least six of the considered 15 classifiers (see Table 2). It was also found that the gene *TSPYL5 *is the most influential and has been chosen by all the considered classifiers. The performance of ANN and SVM was found to be better for the gene subsets {*TSPYL5*, *CA9*}, and {*TSPYL5*, *LIN9*}, respectively. The gene *LIN9 *was found to be important only when using SVM. Similarly, the gene *CA9 *was found to be suitable for the ANN and Logistic Regression along with other genes. An analysis of the experimental results reveals that similar types of classifiers tended to choose the same subset of genes (except one or two different genes in the subset) to obtain the best performance. For instance, Random Forest, Random Forest with bagging and Random Forest with boosting, are essentially similar classifiers with some small variation. All these classifiers chose the gene subset {*TSPYL5*, *DIAPH1*, *ASPM*} for classifying the dataset. However, Random Forest with bagging produced the best accuracy of the three different types of Random Forest considered in this study, adding the gene *NMU *to the common gene subset {*TSPYL5*, *DIAPH1*, *ASPM*}. Thus, a subset of genes used by all classifiers is selected as the important gene subset. Table 3 summarises the classification accuracy for both the individual test set of van 't Veer [3] and for 5 fold cross validation (CV), comparing the use of subsets of the 7 genes, versus the scenario where all 25,000 genes are used.

**Table 3:** Comparison of the weighted accuracy of different classifiers using i) subsets of our 7 genes and ii) all 25,000 genes

**Classifier**	**Subsets of our 7 genes**		**All 25,000 genes**	
	
	Test set (19)	All data (5-fold CV)	Test set (19)	All data (5-fold CV)
C4.5	84.52%	88.49%	79.17%	62.36%

C4.5 with boosting (ADABoost)	91.67%	89.54%	63.10%	62.89%

C4.5 with bagging	84.52%	88.94%	48.81%	63.98%

Naïve Bayes	84.52%	92.13%	50.00%	52.17%

Naïve Bayes with bagging	88.69%	86.82%	50.00%	52.17%

Naïve Bayes with boosting	84.52%	87.65%	50.00%	52.17%

LMT	84.52%	88.11%	77.38%	60.29%

NBTree	84.52%	83.69%	66.07%	58.76%

Random Forest	84.52%	90.59%	66.07%	62.47%

Random Forest with bagging	88.69%	90.59%	73.21%	64.75%

Random Forest with boosting	84.52%	88.48%	66.07%	62.45%

*k*-NN	80.36%	83.00%	63.69%	61.94%

Logistic Regression	81.55%	88.11%	Out of memory*	Out of memory*

ANN	77.38%	83.44%	Out of memory*	Out of memory*

SVM	83.33%	76.23%	63.69%	68.12%

*Our experiments were carried out on a standard Intel Core 2 Duo CPU 2.4 GHz desktop computer running 2 GB of RAM.

#### 6 gene set

Table 4 shows the classification performance of different classifiers on the Ma *et al. *[4] and Loi *et al. *[5] data. Apart from our selection of 6 genes, we have also used the 2 gene biomarker proposed by Ma *et al. *[4] for comparison, bearing in mind that this is a somewhat of a simplification, as the two genes are actually used as a ratio in their study. Our selection of 6 genes is performing much better than the 2 genes on the Ma *et al*. dataset. Of the 15 classifiers, 13 achieve 100% accuracy, whereas the 2 gene biomarker showed a maximum accuracy of only 80% by one classifier. For most classifiers, the 2 gene biomarker showed only 60% to 70% accuracy. When testing on the Loi *et al. *[5] dataset, the performance of the 6 gene set over the 2 gene set is quite strong (9 wins by the 6 gene set, 3 wins by the 2 gene set and 3 draws over the 15 classifiers).

**Table 4:** Comparison of the accuracy of different classifiers using 2 known biomarker genes and our selection of 6 genes on Ma *et al. *and Loi *et al. *data

**Classifier**	**Ma *et al.*data**		**Loi *et al. *data**	
	
	2 genes	6 genes	2 genes	6 genes
C4.5	60.00%	**100%**	75.64%	**80.77%**

C4.5 with boosting (ADABoost)	70.00%	**100%**	66.67%	**82.05%**

C4.5 with bagging	70.00%	**100%**	67.95%	**75.64%**

Naïve Bayes	60.00%	**100%**	74.36%	74.36%

Naïve Bayes with boosting	60.00%	**80.00%**	74.36%	**77.95%**

Naïve Bayes with bagging	60.00%	**100%**	75.64%	75.64%

LMT	70.00%	**100%**	76.92%	**79.49%**

NBTree	80.00%	80.00%	75.64%	**82.05%**

Random Forest	60.00%	**100%**	74.36%	**75.38%**

Random Forest with boosting	70.00%	**100%**	67.95%	**74.36%**

Random Forest with bagging	70.00%	**100%**	**74.36%**	71.79%

*k*-NN	70.00%	**100%**	**73.08%**	71.79%

Logistic Regression	70.00%	**100%**	**76.92%**	74.36%

ANN	60.00%	**100%**	74.36%	**76.67%**

SVM	60.00%	**100%**	74.36%	74.36%

## Discussion

In our multi-classifier voting approach, one selects a subset of genes using an ROC based ranking. This is then followed by a classifier voting phase, to refine this list of genes even further. Our improved performance is achieved due to two factors. First, ROC is a classifier independent method that is not dependent on the standard deviation of the features. Second, the multi-classifier voting gene selection approach produces the best possible combination of genes satisfied by a majority of the classifiers. These two benefits contribute to obtaining a better classification performance for the complete set of unseen datasets. Furthermore, the significant reduction of genes we obtain is another advantage of our approach.

Previous studies that link gene expression profiles to clinical outcomes in breast cancer cases have demonstrated that the potential for distant metastasis and overall survival probability may be attributable to the biological characteristics of the primary tumor at the time of diagnosis [3,35-39]. In particular, a 70-gene expression signature by van 't Veer [3] has proven to be a strong prognostic factor, outperforming all known clinicopathological parameters. The accuracy in distinguishing cases of Poor and Good breast cancer prognosis, provided by the subset of 70 genes selected by van 't Veer [3], was revalidated and confirmed by van de Vijver [39] in a different cohort of patients. However, 70 genes is not a compact set, greatly increasing the expense of developing a clinical predictor. Even the set of 17 genes by Alexe *et al. *[15] is twice as large as our 7 gene set. Our method yielded better accuracy with 7 genes, and that, too, was independent of a specific classifier. Having a compact set of genes is extremely important from a treatment and drug development viewpoint, where clinical and experimental validation is costly and it is vital to restrict the number of hypotheses or targets (genes) to be followed up. We also show, in the comparison of our 6 gene set with the 2 gene set of Ma *et al. *[4] in Table 4 using the Loi *et al. *[5] data, that our approach avoids generating an overly compact geneset that may not generalise well to microarray data from another lab. This is extremely important when attempting to develop a robust predictor in a clinical setting.

We have also compared our best results for the van 't Veer [3] dataset against some well known cancer treatment guidelines (see Table 5). It clearly shows that machine learning approaches are effective technique in classifying breast cancer prognosis.

**Table 5:** Comparison of the weighted accuracy on the test set of the best result from our voting method versus some well known cancer treatment guidelines

**Classifier**	**Weighted accuracy on Test set**
C4.5 with boosting	91.67%

St. Gallen 1998*	68%

NIH 2000*	79%

NPI*	58%

70-genes*	74%

BPIM*	68%

BDIM*	58%

*Results obtained from Gevaert *et al. *[43], where results were provided as number of true positives and true negatives.

### Comparison with other studies

A number of efforts have been made in this direction for breast cancer prognosis but without major success. Ritz [40] combined both genetic and clinical information in a neural network for breast cancer prognosis, but found that the combination did not improve the performance.

Dettling *et al. *[41] applied penalized logistic regression analysis to predict cancer prognosis for the van 't Veer [3] dataset. They found that none of the clinical variables entered the model and concluded that the clinical data did not contain any useful independent information for prediction, given the gene expression profile.

To prognosticate on the breast cancer dataset, Alexe *et al. *[15] applied the Logical Analysis of Data (LAD) tool to analyze microarray data. They identified 17 genes out of 25,000 possible genes that could distinguish patients with Poor or Good prognoses. Amongst the 17 genes the LAD tool identified three and five genes that were associated with Poor and Good prognoses, respectively. Two wholly new classes (defined by similar sets of covering patterns, gene expression ranges, and clinical features) of patients were discovered. It was also demonstrated that the training and test sets of van 't Veer [3] differ in their characteristics. However, this study of Alexe *et al. *was overly specific to the chosen classifier (the LAD tool), as we shall shortly see.

We assessed the classification performance of five different subsets of genes on the van 't Veer [3] dataset. The 231 and 70 genes selected by van 't Veer [3], the set of 17 genes selected by the LAD technique [15], a set of 17 genes selected by ROC (FROC) with Markov blanket [9] and the set of 7 genes selected by our voting approach. For ROC (FROC) with Markov blanket we have used the same parameter for the number of genes to select as given in that paper, namely 50 genes and then using an area between two ROC curves (ABR) value > 20 for the second step, chosen to yield the most competitive performance for the technique. We applied five classification methods used by Alexe *et al. *[15] and our top performing classifier C4.5 with boosting on the gene set of size 4. These classification methods include ANN, SVM, Logistic Regression, *k*-NN, C4.5 decision tree and C4.5 with boosting (see Table 6). Following this, predictive models were constructed for the training set and were tested using the supplied test set of 19 samples.

**Table 6:** Comparison of the classifier performance using i) a variable subset of our 7 genes, ii) a set of 17 genes identified by ROC with Markov Blanket [9], iii) a set of 17 genes identified by LAD [15], iv) a set of 70 genes identified by van 't Veer [3] and v) a set of 231 genes identified by van 't Veer [3]

**Classifier**	**Subsets of our 7 genes**	**Our 7 genes**	**Set of 17 genes **[9]	**Set of 17 genes **[15]	**Set of 70 genes **[3]	**Set of 231 genes **[3]
C4.5 with boosting	91.67%	84.52%	68.42%	59.52%	54.76%	76.19%

C4.5	84.52%	84.52%	68.42%	57.90%*	42.11%*	73.68%*

*k*-NN	80.36%	77.38%	74.21%	63.16%*	63.16%*	78.94%*

Logistic Regression	81.55%	77.38%	73.68%	73.68%*	47.37%*	73.68%*

ANN	77.38%	76.19%	84.21%	84.21%*	42.11%*	73.68%*

SVM	83.33%	76.19%	79.47%	63.16%*	57.90%*	73.68%*

*Results adopted from Alexe *et al. *[15].

It is clear that the weighted accuracy in distinguishing patients with Good and Poor breast cancer prognoses is the highest across all classifiers using the 7 genes selected by our voting approach and is much higher than the models using 17 (by LAD), 70 and 231 genes. Our approach produced much better performance for most classifiers, except for ANN, where using the 17 selected genes of Alexe *et al. *[15] or by using ROC with Markov blanket [9] was better. However, the methodology of Alexe *et al. *incorporated a "selection bias" [42] for finding their subset of 17 genes, since the test set was used. In contrast, our voting approach did not have access to the test set for gene selection. When selecting genes using our voting approach, we used only the training dataset, keeping the test set completely unknown. The performance of the classifiers using the selected 70 and 231 genes by van 't Veer [3] was found to be insignificant compared with that of our approach. Furthermore, 70 or 231 is not a compact set of genes and our voting method can obtain a better accuracy using at most four genes (see Table 2).

Gevaert *et al. *[43] proposed a Bayesian networ *k*-based strategy to treat clinical and microarray data along the same lines as above using the same dataset. A probabilistic model was used because it integrates the data sources in several ways, and explores and documents the model structure and parameters. The concept of a Markov Blanket is used to identify all the variables that shield the class variable from being affected by the rest of the network. However, all the processes are integrated in the classifier, and hence the performance of the system is biased towards the choice of a classifier. Furthermore, the performance of the classifier would depend on the selection of the initial distribution for the model.

### Comparison with other filter approaches

**Figure 1:**
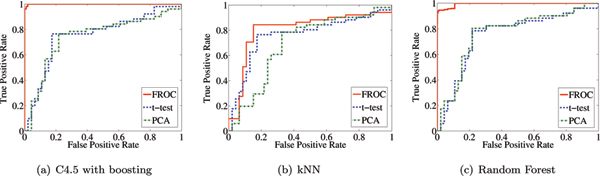
**ROC curves of three classifiers with selected genes using three filter approaches FROC, *t*-test and PCA**. A group of three graphs showing ROC curves for three classifiers with selected genes using three filter approaches FROC, *t*-test and PCA.

Jeffery *et al. *[44] have demonstrated that the ROC is an accurate way to identify differentially regulated genes in a microarray dataset and that it can produce robust classifiers applying 9 feature selection techniques on 9 gene expression datasets. When dealing with datasets that have 15 or more samples, the ROC was shown to be the most accurate. Other filter approaches like *t*-test and Principle Component Analysis (PCA) produce reasonable results, but ROC yields better results (see Figure 1) on the van 't Veer [3] dataset. It is particularly useful for gene expression data, as it is not directly dependent on the standard deviation of the expression of each gene like the *t*-test is, or only on the correlation of each genes like the PCA is. Moreover, unlike PCA, ROC is not sensitive to the scaling of the data.

### Gene set significance tests

It is interesting to consider the results of gene set enrichment analysis (GSEA) [45] on our set of 7 genes obtained by voting, against the other gene sets proposed by van 't Veer *et al. *[3] (size 70 and 231 genes, respectively), Alexe *et al. *[15] (17 genes), and the one we have obtained using Mamitsuka's [9] technique (17 genes). Three out of the five gene sets are enriched in phenotype 1 (i.e. relapse). Of which, Mamitsuka's and our gene sets have an FDR *q*-value equal to 0 and 0.005, with an enrichment score (ES) of 0.78 and 0.80, respectively. Members of the leading edge subset (i.e., tags = 100%, list = 20% and signal = 125%) also indicate that our gene set contains only those genes contributing to the enrichment score, compared to the other gene sets that contain only a fraction of genes contributing to the enrichment score (see Additional File 2).

## Conclusion

We have proposed and implemented a multi-classifier voting approach for gene selection, to effectively classify the prognosis of breast cancer patients using data from two distinct treatment cases. The novelty of our approach is that it can identify a very small number of genes that are predictive across a large range of classifiers. We applied our voting approach to three well-known microarray datasets, related to breast cancer. Experimental analysis demonstrated high prediction accuracies for the gene sets discovered, compared to previous studies. The gene sets discovered were also biologically relevant and had good biological process coverage.

## List of abbreviations used

ROC: Receiver Operating Characteristic; AUC: Area Under the ROC Curve; LMT: Logistic Model Tree; *k*-NN: *k*-Nearest Neighbour; ANN: Artificial Neural Network; SVM: Support Vector Machine; NBTree: Naïve Bayes Tree; CV: Cross Validation; PCA: Principle Component Analysis; GSEA: Gene Set Enrichment Analysis; ES: Enrichment Score; GO: Gene Ontology; KEGG: Kyoto Encyclopedia of Genes and Genomes

## Competing interests

The authors declare that they have no competing interests.

## Authors' contributions

MRH, MMH and KR conceived the design of the ROC ranking and voting algorithm. JB and GM contributed to the experimental design, and the experiments were performed by MMH and MRH. The biological significance was investigated by GM and JWKH and the writing was performed with input from all authors.

## Supplementary Material

Additional file 1 This file contains the rank gene list used in each fold of 5-fold CV, and performance of each fold using the selected genes for different classifier. Click here for file

Additional file 2 This file contains the result of gene set enrichment analysis (GSEA). Click here for file
