# Supplementary material for: A voting approach to identify a small number of highly predictive genes using multiple classifiers
Source: BMC Bioinformatics. 2009 Jan 30;10(Suppl 1):S19. doi: 10.1186/1471-2105-10-S1-S19 (PMC2648737; doi:10.1186/1471-2105-10-S1-S19)
Supplement: Additional file 1 — This file contains the rank gene list used in each fold of 5-fold CV, and performance of each fold using the selected genes for different classifier. [file 1471-2105-10-S1-S19-S1.pdf]

Supplementary Result for "A voting approach to identify small number of highly predictive genes based on multiple classifiers"

Md. Rafiul Hassan*[1], M. Maruf Hossain*[1], James Bailey[1,2], Geoff Macintyre[1,2], Joshua W. K. Ho[3,4], and Kotagiri Ramamohanarao[1,2]

[1]Department of Computer Science and Software Engineering, The University of Melbourne, Victoria 3010, Australia
[2]NICTA Victoria Laboratory, The University of Melbourne, Victoria 3010, Australia
[3]School of Information Technologies, The University of Sydney, NSW 2006, Australia
[4]NICTA, Australian Technology Park, NSW 1430, Australia

Email: Md. Rafiul Hassan*– mrhassan@csse.unimelb.edu.au; M. Maruf Hossain*– hossain@csse.unimelb.edu.au; James Bailey – jbailey@csse.unimelb.edu.au; Geoff Macintyre – gmaci@csse.unimelb.edu.au; Joshua W. K. Ho – joshua@it.usyd.edu.au; Kotagiri Ramamohanarao – rao@csse.unimelb.edu.au;

*Corresponding author

Further discussion

- C4.5 can achieve an average of 88.49% accuracy using at most from 1 to 4 genes for different fold. While in the test set the same classifier used only one gene to achieve a maximum accuracy of 84.52%.
- C4.5 with boosting can achieve an average of 89.54% accuracy using at most from 1 to 5 genes for different fold. While in the test set the same classifier used 4 genes to achieve a maximum accuracy of 91.67%.
- C4.5 with bagging can achieve an average of 88.94% accuracy using at most from 1 to 6 genes for different fold. While in the test set the same classifier used only one gene to achieve a maximum accuracy of 84.52%.
- Naive Bayes can achieve an average of 84.52% accuracy using at most from 1 to 6 genes for different fold. While in the test set the same classifier used only one gene to achieve a maximum accuracy of 84.52%.
- Naive Bayes with bagging can achieve an average of 92.13% accuracy using at most from 1 to 4 genes for different fold. While in the test set the same classifier used 4 genes to achieve a maximum

accuracy of 88.69%.

- Naive Bayes with boosting can achieve an average of 87.65% accuracy using at most from 1 to 4 genes for different fold. While in the test set the same classifier used only one gene to achieve a maximum accuracy of 84.52%.
- LMT can achieve an average of 88.11% accuracy using at most from 2 to 5 genes for different fold. While in the test set the same classifier used only one gene to achieve a maximum accuracy of 84.52%.
- NBTree can achieve an average of 83.69% accuracy using at most from 1 to 6 genes for different fold. While in the test set the same classifier used only one gene to achieve a maximum accuracy of 84.52%.
- Random Forest can achieve an average of 90.59% accuracy using at most from 2 to 6 genes for different fold. While in the test set the same classifier used 4 genes to achieve a maximum accuracy of 84.52%.
- Random Forest with bagging can achieve an average of 90.59% accuracy using at most from 3 to 5 genes for different fold. While in the test set the same classifier used 5 genes to achieve a maximum accuracy of 88.69%.
- Random Forest with boosting can achieve an average of 88.48% accuracy using at most from 2 to 5 genes for different fold. While in the test set the same classifier used 4 genes to achieve a maximum accuracy of 84.52%.
- k-NN can achieve an average of 83.00% accuracy using at most from 1 to 3 genes for different fold. While in the test set the same classifier used only one gene to achieve a maximum accuracy of 80.36%.
- Logistic Regression can achieve an average of 88.11% accuracy using at most from 2 to 5 genes for different fold. While in the test set the same classifier used 4 genes to achieve a maximum accuracy of 81.55%.
- ANN can achieve an average of 89.47% accuracy using at most from 2 to 6 genes for different fold. While in the test set the same classifier used only two genes to achieve a maximum accuracy of 77.38%.
- SVM can achieve an average of 89.54% accuracy using at most from 1 to 5 genes for different fold. While in the test set the same classifier used only two genes to achieve a maximum accuracy of 83.33%.

Classification performance of 5-fold cross validation

To further investigate accuracy, we conducted experiments using the 5-fold CV scheme on van 't Veer [?] dataset. It is worth nothing that our approach consistently performed better for all the classifiers considered in this study, as reflected by the small standard deviation in the performance accuracy for the 5-fold CV (see Table 4). The genes found to be important in the test data were also found to be significant in the 5-fold CV. For example, the gene *TSPYL5* is common in both the test set and all five folds of the 5-fold CV. Similarly, the genes *NMU* and *AGTPBP1* are found to be important in both the test set and in folds four, three and two for the 5-fold CV.

In addition to the above-mentioned genes, some new genes were introduced in each of the folds while using the ROC to select the initial gene sets. However, our voting approach could initially reduce the gene set selected initially to a subset consisting of, at most six genes, and produced a maximum accuracy of 100% (for test fold three and four using Naïve Bayes and its variants, as shown in Table 4) and 92.13% (for the whole 5-fold CV) for an individual classifier. Details of each of the folds, along with the respective classification accuracy for all the 15 classifiers, are given in the supplementary data.

The result for 5-fold CV reveals that our approach is quite generalise and does not vary significantly across different folds of the training data. Furthermore, for most of the classifiers the accuracy for CV is greater than that of the test data. It is logical that the gene subsets selected for each of the folds varies with the variation of the fold. This is due to the selection of the genes from only the training fold, and from keeping the test fold completely unseen. The varying characteristics of genes also support the claim of Alexe *et al.* [?]. For instance, the ANN classifier achieved an average accuracy of 89.47% using at most from two to six genes for different folds, while in the test set, the same classifier used only two genes to achieve a maximum accuracy of 77.38%. A similar trend was also found for all the considered classifiers including C4.5, Naïve Bayes with bagging, LMT, Random Forest and Logistic Regression.

Supplementary results

GeneBank Accession Number	AUC	DAVID Gene Name
NM_005444	0.85034	RCD1 REQUIRED FOR CELL DIFFERENTIATION1 HOMOLOG (S. POMBE)
NM_002268	0.83976	KARYOPHERIN ALPHA 4 (IMPORTIN ALPHA 3)
AA534969	0.83900	HYPOTHETICAL PROTEIN MGC11335
AL080059	0.83749	TSPY-LIKE 5
NM_006681	0.83220	NEUROMEDIN U
AI458074	0.81557	F-BOX PROTEIN 45
NM_000861	0.81406	HISTAMINE RECEPTOR H1
AL157477	0.81104	TRANSMEMBRANE PROTEIN 32
NM_002916	0.81028	REPLICATION FACTOR C (ACTIVATOR 1) 4, 37KDA
NM_017613	0.81028	DOWNSTREAM NEIGHBOR OF SON

Table 1: List of top 10 ranked genes on the training set for Fold 1

Method	Accuracy	Gene Combination
C4.5	89.47%	NM_002268–AL080059, NM_002268–NM_000861
C4.5 with boosting (ADABoost)	84.21%	NM_002268–AI458074, NM_002268–NM_017613
C4.5 with bagging	84.21%	NM_002268–NM_017613–AA534969–NM_000861, NM_002268–NM_017613–AA534969–NM_002916
Naïve Bayes	89.47%	NM_002268–NM_017613
Naïve Bayes with bagging	84.21%	NM_002268–NM_017613–AA534969–AI458074
Naïve Bayes with boosting	84.21%	NM_002268–NM_017613–AA534969–AI458074
LMT	84.21%	NM_002268–NM_017613–AA534969–NM_002916
NBTree	73.68%	NM_002268–NM_017613–AL157477
Random Forest	84.21%	NM_002268–NM_017613
Random Forest with bagging	89.47%	NM_002268–NM_017613–AA534969–AI458074
Random Forest with boosting	84.21%	NM_002268–NM_017613
k-NN	84.21%	NM_002268–NM_017613–NM_000861
Logistic Regression	84.21%	NM_002268–NM_017613–AA534969–AI458074–NM_000861
ANN	89.47%	NM_002268–NM_017613–AA534969–AI458074–NM_005444
SVM	78.95%	NM_002268–NM_017613–AA534969–AI458074–NM_005444

Table 2: Accuracy results on the testset for Fold 1

GeneBank Accession Number	AUC	DAVID Gene Name
AL080059	0.83441	TSPY-LIKE 5
AI123683	0.81362	HYPOTHETICAL LOC388969
AA973313	0.80932	TSPY-LIKE 5
AF073519	0.80932	SMALL EDRK-RICH FACTOR 1A (TELOMERIC)
NM_016017/NM_015984	0.80430	CGI-70 protein (LOC51630)/UBIQUITIN CARBOXYL-TERMINAL HYDROLASE L5
AI419857	0.80215	NUCLEOLAR PROTEIN WITH MIF4G DOMAIN 1
NM_013438	0.79498	UBIQUILIN 1
NM_005744	0.79355	ARIADNE HOMOLOG
AA485242	0.79283	SMALL INDUCIBLE CYTOKINE SUBFAMILY E, MEMBER 1 (ENDOTHELIAL MONOCYTE-A
NM_020120	0.78853	UDP-GLUCOSE CERAMIDE GLUCOSYLTRANSFERASE-LIKE 1

Table 3: List of top 10 ranked genes on the training set for Fold 2

Method	Accuracy	Gene Combination
C4.5	96.67%	NM_016017/NM_015984–AA973313–NM_013438–AF073519
C4.5 with boosting (ADABoost)	100.00%	NM_016017/NM_015984–AA973313–NM_013438–AF073519–NM_020120
C4.5 with bagging	100.00%	NM_016017/NM_015984–AA973313–NM_013438–AF073519
Naïve Bayes	86.67%	NM_016017/NM_015984–AI419857, NM_016017/NM_015984–AA485242
Naïve Bayes with bagging	76.67%	NM_005744
Naïve Bayes with boosting	76.67%	NM_005744
LMT	100%	NM_016017/NM_015984–AA973313–NM_013438–AF073519–NM_005744
NBTree	96.67%	NM_016017/NM_015984–AA973313–NM_013438–AF073519–NM_005744–AI419857
Random Forest	100%	NM_016017/NM_015984–AA973313–NM_013438–AF073519–NM_005744, NM_016017/NM_015984–AA973313–NM_013438–AF073519–NM_020120
Random Forest with bagging	100%	NM_016017/NM_015984–AA973313–NM_013438–AF073519
Random Forest with boosting	100%	NM_016017/NM_015984–AA973313–NM_013438–AF073519–NM_020120
k-NN	93.33%	NM_016017/NM_015984–AA973313
Logistic Regression	100%	NM_016017/NM_015984–AA973313–NM_013438–AF073519–NM_005744
ANN	76.67%	NM_016017/NM_015984–NM_005744
SVM	70%	AF073519

Table 4: Accuracy results on the testset for Fold 2

GeneBank Accession Number	AUC	DAVID Gene Name
NM_006681	0.82464	NEUROMEDIN U
NM_005744	0.82101	ARIADNE HOMOLOG
NM_005192	0.81159	CYCLIN-DEPENDENT KINASE INHIBITOR 3 (CDK2-ASSOCIATED DUAL SPECIFICITY)
NM_020120	0.80797	UDP-GLUCOSE CERAMIDE GLUCOSYLTRANSFERASE-LIKE 1
AA748494	0.79638	HYPOTHETICAL PROTEIN FLJ10517
AL049397	0.79058	CHROMOSOME 1 OPEN READING FRAME 121
NM_016076	0.78768	CHROMOSOME 1 OPEN READING FRAME 121
AL080059	0.78623	TSPY-LIKE 5
NM_014547	0.78623	TROPOMODULIN 3 (UBIQUITOUS)
AF161553	0.78406	INFLUENZA VIRUS NS1A BINDING PROTEIN

Table 5: List of top 10 ranked genes on the training set for Fold 3

Method	Accuracy	Gene Combination
C4.5	89.47%	NM_006681
C4.5 with boosting (ADABoost)	94.74%	NM_006681–NM_005192
C4.5 with bagging	89.47%	NM_006681
Naïve Bayes	100%	NM_005744, NM_005192, AA748494, AL049397, NM_016076, NM_014547, AF161553
Naïve Bayes with bagging	100%	NM_005744, NM_005192, AA748494, AL049397, NM_016076, NM_014547, AF161553
Naïve Bayes with boosting	100%	NM_005744, NM_005192, AA748494, AL049397, NM_016076, NM_014547, AF161553
LMT	89.47%	NM_006681–NM_020120–NM_005744–NM_005192–AL049397
NBTree	89.47%	NM_006681
Random Forest	94.74%	NM_006681–NM_020120–NM_005744–NM_005192–NM_014547
Random Forest with bagging	89.47%	NM_006681–NM_020120–NM_005744
Random Forest with boosting	89.47%	NM_006681–NM_020120–NM_005744–NM_005192, NM_006681–NM_020120–NM_005744–AL080059
k-NN	84.21%	NM_006681–NM_020120–AL080059
Logistic Regression	89.47%	NM_006681–NM_020120–NM_005744–NM_005192–AL049397, NM_006681–NM_020120–NM_005744–NM_005192–NM_014547
ANN	89.47%	NM_006681–NM_020120
SVM	78.95%	NM_006681–NM_020120

Table 6: Accuracy results on the testset for Fold 3

GeneBank Accession Number	AUC	DAVID Gene Name
AL080059	0.80145	TSPY-LIKE 5
NM_005217	0.77899	DEFENSIN, ALPHA 1
AA973313	0.77391	TSPY-LIKE 5
AA830802	0.77029	ATP/GTP BINDING PROTEIN 1
NM_016017/NM_015984	0.76812	CGI-70 protein (LOC51630)/UBIQUITIN CARBOXYL-TERMINAL HYDROLASE L5
NM_006681	0.76522	NEUROMEDIN U
NM_004994	0.75870	MATRIX METALLOPEPTIDASE 9 (GELATINASE B, 92KDA GELATINASE)
NM_016448	0.75652	DENTICLELESS HOMOLOG (DROSOPHILA)
AI813331	0.75435	DIAPHANOUS HOMOLOG 3 (DROSOPHILA)
NM_004702	0.75217	CYCLIN E2 (CCNE2), TRANSCRIPT VARIANT 3 (PERMANENTLY SUPPRESSED)

Table 7: List of top 10 ranked genes on the training set for Fold 4

Method	Accuracy	Gene Combination
C4.5	89.47%	NM_005217–NM_004702–AL080059
C4.5 with boosting (ADABoost)	84.21%	NM_005217
C4.5 with bagging	89.47%	NM_005217–NM_004702–AL080059–AI813331–NM_016017/NM_015984–NM_016448
Naïve Bayes	100%	NM_005217, AA973313, NM_006681, NM_004994, AI813331, NM_004702
Naïve Bayes with bagging	100%	NM_005217, AA973313, NM_006681, NM_004994, AI813331, NM_004702
Naïve Bayes with boosting	100%	NM_005217, AA973313, NM_006681, NM_004994, AI813331, NM_004702
LMT	89.47%	NM_005217–NM_004702–AA830802
NBTree	84.21%	NM_005217
Random Forest	89.47%	NM_005217–NM_004702–AL080059–AI813331–NM_016017/NM_015984–NM_004994
Random Forest with bagging	89.47%	NM_005217–NM_004702–AL080059–AI813331–NM_016017/NM_015984
Random Forest with boosting	84.21%	NM_005217–NM_004702–NM_016017/NM_015984
k-NN	84.21%	NM_005217–NM_004702–NM_016017/NM_015984
Logistic Regression	89.47%	NM_005217–NM_004702–AL080059–NM_006681
ANN	84.21%	NM_005217–NM_004702–AL080059–AI813331–NM_016017/NM_015984–AA830802
SVM	84.21%	NM_005217–NM_004702–AL080059–AI813331–AA830802

Table 8: Accuracy results on the testset for Fold 4

GeneBank Accession Number	AUC	DAVID Gene Name
AL080059	0.82283	TSPY-LIKE 5
AA973313	0.81022	TSPY-LIKE 5
NM_006544	0.80182	EXOCYST COMPLEX COMPONENT 5
NM_001216	0.80182	CARBONIC ANHYDRASE IX
NM_006681	0.80042	NEUROMEDIN U
AA830802	0.78711	ATP/GTP BINDING PROTEIN 1
AA834945	0.78571	SIMILAR TO NUCLEASE SENSITIVE ELEMENT BINDING PROTEIN 1
AI377418	0.77521	INTIMAL THICKNESS-RELATED RECEPTOR
NM_012479	0.77381	TYROSINE 3-MONOOXYGENASE/TRYPTOPHAN 5-MONOOXYGENASE ACTIVATION PROTEIN
NM_005219	0.77171	DIAPHANOUS HOMOLOG 1 (DROSOPHILA)

Table 9: List of top 10 ranked genes on the training set for Fold 5

Method	Accuracy	Gene Combination
C4.5	77.38%	AL080059–NM_005219
C4.5 with boosting (ADABoost)	84.52%	AL080059–NM_005219–NM_012479
C4.5 with bagging	81.55%	AL080059–NM_005219–NM_006681
Naïve Bayes	84.52%	AL080059–NM_005219–NM_012479–AA973313–NM_006681–AA830802
Naïve Bayes with bagging	73.21%	AL080059–NM_001216
Naïve Bayes with boosting	77.38%	AL080059–NM_001216
LMT	77.38%	AL080059–NM_001216
NBTree	74.40%	AL080059–NM_005219–NM_012479–AA973313–NM_006681–AA834945
Random Forest	84.52%	AL080059–NM_005219–AA973313
Random Forest with bagging	84.52%	AL080059–NM_005219–NM_012479
Random Forest with boosting	84.52%	AL080059–NM_005219–NM_012479
k-NN	69.05%	NM_006681
Logistic Regression	77.38%	AL080059–NM_001216
ANN	77.38%	AL080059–NM_001216
SVM	69.05%	AL080059–NM_001216

Table 10: Accuracy results on the testset for Fold 5

Classifier	Fold 1	Fold 2	Fold 3	Fold 4	Fold 5	Average
C4.5	89.47%	96.67%	89.47%	89.47%	77.38%	$88.49 \pm 0.069503\%$
C4.5 with boosting (ADABoost)	84.21%	100.00%	94.74%	84.21%	84.52%	$89.54 \pm 0.073904\%$
C4.5 with bagging	84.21%	100.00%	89.47%	89.47%	81.55%	$88.94 \pm 0.070688\%$
Naïve Bayes	89.47%	86.67%	100%	100%	84.52%	$92.13 \pm 0.073938\%$
Naïve Bayes with bagging	84.21%	76.67%	100%	100%	73.21%	$86.82 \pm 0.126737\%$
Naïve Bayes with boosting	84.21%	76.67%	100%	100%	77.38%	$87.65 \pm 0.116502\%$
LMT	84.21%	100%	89.47%	89.47%	77.38%	$88.11 \pm 0.082978\%$
NBTree	73.68%	96.67%	89.47%	84.21%	74.40%	$83.69 \pm 0.098573\%$
Random Forest	84.21%	100%	94.74%	89.47%	84.52%	$90.59 \pm 0.067929\%$
Random Forest with bagging	89.47%	100%	89.47%	89.47%	84.52%	$90.59 \pm 0.056823\%$
Random Forest with boosting	84.21%	100%	89.47%	84.21%	84.52%	$88.48 \pm 0.068161\%$
k-NN	84.21%	93.33%	84.21%	84.21%	69.05%	$83.00 \pm 0.087422\%$
Logistic Regression	84.21%	100%	89.47%	89.47%	77.38%	$88.11 \pm 0.082978\%$
ANN	89.47%	76.67%	89.47%	84.21%	77.38%	$83.44 \pm 0.062424\%$
SVM	78.95%	70%	78.95%	84.21%	69.05%	$76.23 \pm 0.06497\%$

Table 11: Classification performance for 5-fold CV
