# Supplementary material for: A voting approach to identify a small number of highly predictive genes using multiple classifiers
Source: BMC Bioinformatics. 2009 Jan 30;10(Suppl 1):S19. doi: 10.1186/1471-2105-10-S1-S19 (PMC2648737; doi:10.1186/1471-2105-10-S1-S19)
Supplement: Additional file 2 — This file contains the result of gene set enrichment analysis (GSEA). [file 1471-2105-10-S1-S19-S2.zip › ALEXE.html]

Details for gene set ALEXE[GSEA]

|  || Dataset | dataset.phenotype.cls #relapse\_versus\_non-relapse.phenotype.cls #relapse\_versus\_non-relapse\_repos |
| Phenotype | phenotype.cls#relapse\_versus\_non-relapse\_repos |
| Upregulated in class | 0 |
| GeneSet | ALEXE |
| Enrichment Score (ES) | -0.7333945 |
| Normalized Enrichment Score (NES) | -1.8589017 |
| Nominal p-value | 0.0 |
| FDR q-value | 0.017006803 |
| FWER p-Value | 0.04 |
Table: GSEA Results Summary

  

Fig 1: Enrichment plot: ALEXE      
 Profile of the Running ES Score & Positions of GeneSet Members on the Rank Ordered List

  

| PROBE | DESCRIPTION (from dataset) | GENE SYMBOL | GENE\_TITLE | RANK IN GENE LIST | RANK METRIC SCORE | RUNNING ES | CORE ENRICHMENT || 1 | AL080059 | na | AL080059 Entrez,  Source | NULL | 0 | 6.260 | 0.0588 | No |
| 2 | Contig55574\_RC | na | CONTIG55574\_RC Entrez,  Source | NULL | 466 | 2.608 | 0.0986 | No |
| 3 | NM\_001756 | na | NM\_001756 Entrez,  Source | NULL | 1796 | 1.601 | 0.1031 | No |
| 4 | Contig38451\_RC | na | CONTIG38451\_RC Entrez,  Source | NULL | 20452 | -0.829 | -0.6006 | No |
| 5 | Contig15031\_RC | na | CONTIG15031\_RC Entrez,  Source | NULL | 23702 | -2.291 | -0.6746 | Yes |
| 6 | Contig63102\_RC | na | CONTIG63102\_RC Entrez,  Source | NULL | 23747 | -2.338 | -0.6175 | Yes |
| 7 | AL110129 | na | AL110129 Entrez,  Source | NULL | 24000 | -2.595 | -0.5690 | Yes |
| 8 | Contig42421\_RC | na | CONTIG42421\_RC Entrez,  Source | NULL | 24092 | -2.719 | -0.5139 | Yes |
| 9 | Contig41383\_RC | na | CONTIG41383\_RC Entrez,  Source | NULL | 24128 | -2.765 | -0.4565 | Yes |
| 10 | Contig37063\_RC | na | CONTIG37063\_RC Entrez,  Source | NULL | 24148 | -2.799 | -0.3985 | Yes |
| 11 | AB033007 | na | AB033007 Entrez,  Source | NULL | 24374 | -3.382 | -0.3489 | Yes |
| 12 | AL049689 | na | AL049689 Entrez,  Source | NULL | 24415 | -3.584 | -0.2917 | Yes |
| 13 | NM\_003748 | na | NM\_003748 Entrez,  Source | NULL | 24421 | -3.624 | -0.2330 | Yes |
| 14 | AF148505 | na | AF148505 Entrez,  Source | NULL | 24431 | -3.691 | -0.1746 | Yes |
| 15 | Contig65439 | na | CONTIG65439 Entrez,  Source | NULL | 24432 | -3.699 | -0.1158 | Yes |
| 16 | NM\_001661 | na | NM\_001661 Entrez,  Source | NULL | 24445 | -3.820 | -0.0574 | Yes |
| 17 | NM\_020974 | na | NM\_020974 Entrez,  Source | NULL | 24480 | -4.823 | 0.0000 | Yes |
Table: GSEA details [plain text format]

  

Fig 2: ALEXE      
 Blue-Pink O' Gram in the Space of the Analyzed GeneSet

  

Fig 3: ALEXE: Random ES distribution      
 Gene set null distribution of ES for **ALEXE**

  
