# Supplementary material for: A voting approach to identify a small number of highly predictive genes using multiple classifiers
Source: BMC Bioinformatics. 2009 Jan 30;10(Suppl 1):S19. doi: 10.1186/1471-2105-10-S1-S19 (PMC2648737; doi:10.1186/1471-2105-10-S1-S19)
Supplement: Additional file 2 — This file contains the result of gene set enrichment analysis (GSEA). [file 1471-2105-10-S1-S19-S2.zip › gsea_report_for_0_1217226852592.html]

Report for 0 1217226852592 [GSEA]

| GS  follow link to MSigDB | GS DETAILS | SIZE | ES | NES | NOM p-val | FDR q-val | FWER p-val | RANK AT MAX | LEADING EDGE || 1 | VANT VEER 70 | Details ... | 70 | -0.93 | -2.54 | 0.000 | 0.000 | 0.000 | 812 | tags=96%, list=3%, signal=99% |
| 2 | ALEXE | Details ... | 17 | -0.73 | -1.86 | 0.000 | 0.017 | 0.040 | 780 | tags=76%, list=3%, signal=79% |
Table: Gene sets enriched in phenotype **0 (51 samples)**[plain text format]****

  
