# Supplementary material for: A voting approach to identify a small number of highly predictive genes using multiple classifiers
Source: BMC Bioinformatics. 2009 Jan 30;10(Suppl 1):S19. doi: 10.1186/1471-2105-10-S1-S19 (PMC2648737; doi:10.1186/1471-2105-10-S1-S19)
Supplement: Additional file 2 — This file contains the result of gene set enrichment analysis (GSEA). [file 1471-2105-10-S1-S19-S2.zip › gsea_report_for_1_1217226852592.html]

Report for 1 1217226852592 [GSEA]

| GS  follow link to MSigDB | GS DETAILS | SIZE | ES | NES | NOM p-val | FDR q-val | FWER p-val | RANK AT MAX | LEADING EDGE || 1 | MAMITSUKA | Details ... | 17 | 0.78 | 2.83 | 0.000 | 0.000 | 0.000 | 5343 | tags=100%, list=22%, signal=128% |
| 2 | VOTING | Details ... | 7 | 0.80 | 2.12 | 0.000 | 0.005 | 0.010 | 4943 | tags=100%, list=20%, signal=125% |
| 3 | VANT VEER 231 | Details ... | 231 | 0.58 | 1.82 | 0.000 | 0.009 | 0.020 | 1505 | tags=64%, list=6%, signal=67% |
Table: Gene sets enriched in phenotype **1 (46 samples)**[plain text format]****

  
