# Supplementary material for: A voting approach to identify a small number of highly predictive genes using multiple classifiers
Source: BMC Bioinformatics. 2009 Jan 30;10(Suppl 1):S19. doi: 10.1186/1471-2105-10-S1-S19 (PMC2648737; doi:10.1186/1471-2105-10-S1-S19)
Supplement: Additional file 2 — This file contains the result of gene set enrichment analysis (GSEA). [file 1471-2105-10-S1-S19-S2.zip › heat_map_corr_plot.html]

Heat map and correlation plot for dataset.phenotype.cls#relapse\_versus\_non-relapse  

Fig 1: heat\_map      
 Heat Map of the top 50 features for each phenotype in dataset.phenotype.cls#relapse\_versus\_non-relapse

  
  

Fig 2: Ranked Gene List Correlation Profile      
 Ranked list correlations for dataset.phenotype.cls#relapse\_versus\_non-relapse

  
  
    
