# Supplementary material for: A voting approach to identify a small number of highly predictive genes using multiple classifiers
Source: BMC Bioinformatics. 2009 Jan 30;10(Suppl 1):S19. doi: 10.1186/1471-2105-10-S1-S19 (PMC2648737; doi:10.1186/1471-2105-10-S1-S19)
Supplement: Additional file 2 — This file contains the result of gene set enrichment analysis (GSEA). [file 1471-2105-10-S1-S19-S2.zip › index.html]

Index for xtools.gsea.Gsea my\_analysis.Gsea.1217226852592

### GSEA Report for Dataset dataset

#### Enrichment in phenotype: **1 (46 samples)**

- 3 / 5 gene sets are upregulated in phenotype **1**- 3 gene sets are significant at FDR < 25%- 3 gene sets are significantly enriched at nominal pvalue < 1%- 3 gene sets are significantly enriched at nominal pvalue < 5%- Snapshot of enrichment results- Detailed enrichment results in html format- Detailed enrichment results in excel format (tab delimited text)- Guide to interpret results

#### Enrichment in phenotype: **0 (51 samples)**

- 2 / 5 gene sets are upregulated in phenotype **0**- 2 gene sets are significantly enriched at FDR < 25%- 2 gene sets are significantly enriched at nominal pvalue < 1%- 2 gene sets are significantly enriched at nominal pvalue < 5%- Snapshot of enrichment results- Detailed enrichment results in html format- Detailed enrichment results in excel format (tab delimited text)- Guide to interpret results

#### Dataset details

- The dataset has 24481 features (genes)- No probe set => gene symbol collapsing was requested, so all 24481 features were used

#### Gene set details

- Gene set size filters (min=7, max=231) resulted in filtering out 0 / 5 gene sets- The remaining 5 gene sets were used in the analysis- List of gene sets used and their sizes (restricted to features in the specified dataset)

#### Gene markers for the **1** *versus* **0** comparison

- The dataset has 24481 features (genes)- # of markers for phenotype **1**: 17210 (70.3% ) with correlation area 69.9%- # of markers for phenotype **0**: 7271 (29.7% ) with correlation area 30.1%- Detailed rank ordered gene list for all features in the dataset- Heat map and gene list correlation  profile for all features in the dataset- Buttefly plot of significant genes

#### Global statistics and plots

- Plot of p-values *vs.* NES- Global ES histogram

#### Other

- Parameters used for this analysis

---

Report: my\_analysis.Gsea.1217226852592.rpt   by user: Maruf

xtools.gsea.Gsea [Mon, Jul 28, '08 4 PM 34]

Website: www.broad.mit.edu/GSEA
Questions & Suggestions: Email
