# Supplementary material for: A voting approach to identify a small number of highly predictive genes using multiple classifiers
Source: BMC Bioinformatics. 2009 Jan 30;10(Suppl 1):S19. doi: 10.1186/1471-2105-10-S1-S19 (PMC2648737; doi:10.1186/1471-2105-10-S1-S19)
Supplement: Additional file 2 — This file contains the result of gene set enrichment analysis (GSEA). [file 1471-2105-10-S1-S19-S2.zip › MAMITSUKA.html]

Details for gene set MAMITSUKA[GSEA]

|  || Dataset | dataset.phenotype.cls #relapse\_versus\_non-relapse.phenotype.cls #relapse\_versus\_non-relapse\_repos |
| Phenotype | phenotype.cls#relapse\_versus\_non-relapse\_repos |
| Upregulated in class | 1 |
| GeneSet | MAMITSUKA |
| Enrichment Score (ES) | 0.7822515 |
| Normalized Enrichment Score (NES) | 2.8310926 |
| Nominal p-value | 0.0 |
| FDR q-value | 0.0 |
| FWER p-Value | 0.0 |
Table: GSEA Results Summary

  

Fig 1: Enrichment plot: MAMITSUKA      
 Profile of the Running ES Score & Positions of GeneSet Members on the Rank Ordered List

  

| PROBE | DESCRIPTION (from dataset) | GENE SYMBOL | GENE\_TITLE | RANK IN GENE LIST | RANK METRIC SCORE | RUNNING ES | CORE ENRICHMENT || 1 | NM\_016359 | na | NM\_016359 Entrez,  Source | NULL | 7 | 4.404 | 0.0585 | Yes |
| 2 | Contig55725\_RC | na | CONTIG55725\_RC Entrez,  Source | NULL | 8 | 4.323 | 0.1174 | Yes |
| 3 | NM\_003875 | na | NM\_003875 Entrez,  Source | NULL | 9 | 4.320 | 0.1762 | Yes |
| 4 | Contig54260\_RC | na | CONTIG54260\_RC Entrez,  Source | NULL | 18 | 4.112 | 0.2347 | Yes |
| 5 | Contig53488 | na | CONTIG53488 Entrez,  Source | NULL | 33 | 3.975 | 0.2929 | Yes |
| 6 | Contig53223 | na | CONTIG53223 Entrez,  Source | NULL | 38 | 3.883 | 0.3516 | Yes |
| 7 | AF073519 | na | AF073519 Entrez,  Source | NULL | 57 | 3.750 | 0.4097 | Yes |
| 8 | NM\_014176 | na | NM\_014176 Entrez,  Source | NULL | 137 | 3.330 | 0.4653 | Yes |
| 9 | AL035297 | na | AL035297 Entrez,  Source | NULL | 140 | 3.319 | 0.5240 | Yes |
| 10 | NM\_018455 | na | NM\_018455 Entrez,  Source | NULL | 153 | 3.274 | 0.5823 | Yes |
| 11 | Contig34952 | na | CONTIG34952 Entrez,  Source | NULL | 4681 | 1.115 | 0.4561 | Yes |
| 12 | NM\_021000 | na | NM\_021000 Entrez,  Source | NULL | 4820 | 1.084 | 0.5093 | Yes |
| 13 | NM\_005192 | na | NM\_005192 Entrez,  Source | NULL | 4836 | 1.082 | 0.5675 | Yes |
| 14 | NM\_016017 | na | NM\_016017 Entrez,  Source | NULL | 4867 | 1.077 | 0.6251 | Yes |
| 15 | Contig64861\_RC | na | CONTIG64861\_RC Entrez,  Source | NULL | 5001 | 1.059 | 0.6785 | Yes |
| 16 | Contig55189\_RC | na | CONTIG55189\_RC Entrez,  Source | NULL | 5084 | 1.052 | 0.7340 | Yes |
| 17 | NM\_006544 | na | NM\_006544 Entrez,  Source | NULL | 5343 | 1.039 | 0.7823 | Yes |
Table: GSEA details [plain text format]

  

Fig 2: MAMITSUKA      
 Blue-Pink O' Gram in the Space of the Analyzed GeneSet

  

Fig 3: MAMITSUKA: Random ES distribution      
 Gene set null distribution of ES for **MAMITSUKA**

  
