# Supplementary material for: A voting approach to identify a small number of highly predictive genes using multiple classifiers
Source: BMC Bioinformatics. 2009 Jan 30;10(Suppl 1):S19. doi: 10.1186/1471-2105-10-S1-S19 (PMC2648737; doi:10.1186/1471-2105-10-S1-S19)
Supplement: Additional file 2 — This file contains the result of gene set enrichment analysis (GSEA). [file 1471-2105-10-S1-S19-S2.zip › neg_snapshot.html]

Snapshot of 2 enrichment plots

|  |  |
| --- | --- |
|  |  |
Table: Snapshot of enrichment results

  
