# Supplementary material for: A voting approach to identify a small number of highly predictive genes using multiple classifiers
Source: BMC Bioinformatics. 2009 Jan 30;10(Suppl 1):S19. doi: 10.1186/1471-2105-10-S1-S19 (PMC2648737; doi:10.1186/1471-2105-10-S1-S19)
Supplement: Additional file 2 — This file contains the result of gene set enrichment analysis (GSEA). [file 1471-2105-10-S1-S19-S2.zip › VANT VEER 231.html]

Details for gene set VANT VEER 231[GSEA]

|  || Dataset | dataset.phenotype.cls #relapse\_versus\_non-relapse.phenotype.cls #relapse\_versus\_non-relapse\_repos |
| Phenotype | phenotype.cls#relapse\_versus\_non-relapse\_repos |
| Upregulated in class | 1 |
| GeneSet | VANT VEER 231 |
| Enrichment Score (ES) | 0.5803224 |
| Normalized Enrichment Score (NES) | 1.8224893 |
| Nominal p-value | 0.0 |
| FDR q-value | 0.008503402 |
| FWER p-Value | 0.02 |
Table: GSEA Results Summary

  

Fig 1: Enrichment plot: VANT VEER 231      
 Profile of the Running ES Score & Positions of GeneSet Members on the Rank Ordered List

  

| PROBE | DESCRIPTION (from dataset) | GENE SYMBOL | GENE\_TITLE | RANK IN GENE LIST | RANK METRIC SCORE | RUNNING ES | CORE ENRICHMENT || 1 | AL080059 | na | AL080059 Entrez,  Source | NULL | 0 | 6.260 | 0.0043 | Yes |
| 2 | NM\_016448 | na | NM\_016448 Entrez,  Source | NULL | 1 | 4.783 | 0.0087 | Yes |
| 3 | NM\_001168 | na | NM\_001168 Entrez,  Source | NULL | 6 | 4.442 | 0.0128 | Yes |
| 4 | NM\_016359 | na | NM\_016359 Entrez,  Source | NULL | 7 | 4.404 | 0.0172 | Yes |
| 5 | Contig55725\_RC | na | CONTIG55725\_RC Entrez,  Source | NULL | 8 | 4.323 | 0.0215 | Yes |
| 6 | NM\_003875 | na | NM\_003875 Entrez,  Source | NULL | 9 | 4.320 | 0.0258 | Yes |
| 7 | NM\_006115 | na | NM\_006115 Entrez,  Source | NULL | 10 | 4.297 | 0.0301 | Yes |
| 8 | Contig46218\_RC | na | CONTIG46218\_RC Entrez,  Source | NULL | 12 | 4.265 | 0.0344 | Yes |
| 9 | NM\_002811 | na | NM\_002811 Entrez,  Source | NULL | 13 | 4.261 | 0.0388 | Yes |
| 10 | NM\_003981 | na | NM\_003981 Entrez,  Source | NULL | 14 | 4.230 | 0.0431 | Yes |
| 11 | NM\_004504 | na | NM\_004504 Entrez,  Source | NULL | 16 | 4.169 | 0.0474 | Yes |
| 12 | Contig28552\_RC | na | CONTIG28552\_RC Entrez,  Source | NULL | 17 | 4.134 | 0.0517 | Yes |
| 13 | NM\_004701 | na | NM\_004701 Entrez,  Source | NULL | 19 | 4.086 | 0.0560 | Yes |
| 14 | Contig41413\_RC | na | CONTIG41413\_RC Entrez,  Source | NULL | 22 | 4.056 | 0.0602 | Yes |
| 15 | NM\_005915 | na | NM\_005915 Entrez,  Source | NULL | 25 | 4.030 | 0.0645 | Yes |
| 16 | NM\_002358 | na | NM\_002358 Entrez,  Source | NULL | 27 | 4.008 | 0.0688 | Yes |
| 17 | NM\_020188 | na | NM\_020188 Entrez,  Source | NULL | 29 | 4.002 | 0.0731 | Yes |
| 18 | NM\_001809 | na | NM\_001809 Entrez,  Source | NULL | 31 | 3.984 | 0.0773 | Yes |
| 19 | NM\_018354 | na | NM\_018354 Entrez,  Source | NULL | 32 | 3.983 | 0.0817 | Yes |
| 20 | AF161553 | na | AF161553 Entrez,  Source | NULL | 35 | 3.952 | 0.0859 | Yes |
| 21 | NM\_018410 | na | NM\_018410 Entrez,  Source | NULL | 36 | 3.946 | 0.0902 | Yes |
| 22 | NM\_015984 | na | NM\_015984 Entrez,  Source | NULL | 39 | 3.880 | 0.0945 | Yes |
| 23 | NM\_015434 | na | NM\_015434 Entrez,  Source | NULL | 40 | 3.876 | 0.0988 | Yes |
| 24 | NM\_004994 | na | NM\_004994 Entrez,  Source | NULL | 41 | 3.869 | 0.1032 | Yes |
| 25 | NM\_018265 | na | NM\_018265 Entrez,  Source | NULL | 43 | 3.848 | 0.1074 | Yes |
| 26 | AF052162 | na | AF052162 Entrez,  Source | NULL | 45 | 3.833 | 0.1117 | Yes |
| 27 | X05610 | na | X05610 Entrez,  Source | NULL | 46 | 3.831 | 0.1161 | Yes |
| 28 | NM\_014321 | na | NM\_014321 Entrez,  Source | NULL | 48 | 3.811 | 0.1203 | Yes |
| 29 | NM\_005342 | na | NM\_005342 Entrez,  Source | NULL | 52 | 3.787 | 0.1246 | Yes |
| 30 | NM\_000291 | na | NM\_000291 Entrez,  Source | NULL | 54 | 3.755 | 0.1288 | Yes |
| 31 | NM\_001673 | na | NM\_001673 Entrez,  Source | NULL | 55 | 3.752 | 0.1332 | Yes |
| 32 | AF073519 | na | AF073519 Entrez,  Source | NULL | 57 | 3.750 | 0.1375 | Yes |
| 33 | NM\_006101 | na | NM\_006101 Entrez,  Source | NULL | 60 | 3.725 | 0.1417 | Yes |
| 34 | NM\_000127 | na | NM\_000127 Entrez,  Source | NULL | 61 | 3.694 | 0.1460 | Yes |
| 35 | NM\_002808 | na | NM\_002808 Entrez,  Source | NULL | 62 | 3.693 | 0.1504 | Yes |
| 36 | NM\_003258 | na | NM\_003258 Entrez,  Source | NULL | 63 | 3.688 | 0.1547 | Yes |
| 37 | U96131 | na | U96131 Entrez,  Source | NULL | 65 | 3.685 | 0.1590 | Yes |
| 38 | NM\_003600 | na | NM\_003600 Entrez,  Source | NULL | 66 | 3.671 | 0.1633 | Yes |
| 39 | NM\_020166 | na | NM\_020166 Entrez,  Source | NULL | 68 | 3.653 | 0.1676 | Yes |
| 40 | NM\_003662 | na | NM\_003662 Entrez,  Source | NULL | 69 | 3.640 | 0.1719 | Yes |
| 41 | Contig25343\_RC | na | CONTIG25343\_RC Entrez,  Source | NULL | 70 | 3.634 | 0.1763 | Yes |
| 42 | NM\_002916 | na | NM\_002916 Entrez,  Source | NULL | 71 | 3.631 | 0.1806 | Yes |
| 43 | Contig38288\_RC | na | CONTIG38288\_RC Entrez,  Source | NULL | 73 | 3.629 | 0.1849 | Yes |
| 44 | NM\_001905 | na | NM\_001905 Entrez,  Source | NULL | 75 | 3.621 | 0.1892 | Yes |
| 45 | AA555029\_RC | na | AA555029\_RC Entrez,  Source | NULL | 76 | 3.598 | 0.1935 | Yes |
| 46 | Contig32185\_RC | na | CONTIG32185\_RC Entrez,  Source | NULL | 78 | 3.584 | 0.1978 | Yes |
| 47 | NM\_006201 | na | NM\_006201 Entrez,  Source | NULL | 82 | 3.569 | 0.2020 | Yes |
| 48 | NM\_018136 | na | NM\_018136 Entrez,  Source | NULL | 91 | 3.509 | 0.2060 | Yes |
| 49 | Contig24252\_RC | na | CONTIG24252\_RC Entrez,  Source | NULL | 92 | 3.500 | 0.2103 | Yes |
| 50 | NM\_004456 | na | NM\_004456 Entrez,  Source | NULL | 95 | 3.493 | 0.2146 | Yes |
| 51 | Contig35251\_RC | na | CONTIG35251\_RC Entrez,  Source | NULL | 96 | 3.491 | 0.2189 | Yes |
| 52 | NM\_014875 | na | NM\_014875 Entrez,  Source | NULL | 99 | 3.481 | 0.2231 | Yes |
| 53 | NM\_001216 | na | NM\_001216 Entrez,  Source | NULL | 114 | 3.408 | 0.2269 | Yes |
| 54 | AL050021 | na | AL050021 Entrez,  Source | NULL | 118 | 3.388 | 0.2311 | Yes |
| 55 | NM\_016577 | na | NM\_016577 Entrez,  Source | NULL | 125 | 3.363 | 0.2352 | Yes |
| 56 | NM\_005563 | na | NM\_005563 Entrez,  Source | NULL | 126 | 3.356 | 0.2395 | Yes |
| 57 | NM\_005196 | na | NM\_005196 Entrez,  Source | NULL | 128 | 3.353 | 0.2438 | Yes |
| 58 | Contig25991 | na | CONTIG25991 Entrez,  Source | NULL | 131 | 3.348 | 0.2480 | Yes |
| 59 | Contig51464\_RC | na | CONTIG51464\_RC Entrez,  Source | NULL | 133 | 3.341 | 0.2523 | Yes |
| 60 | NM\_003878 | na | NM\_003878 Entrez,  Source | NULL | 135 | 3.336 | 0.2566 | Yes |
| 61 | AF055033 | na | AF055033 Entrez,  Source | NULL | 136 | 3.333 | 0.2609 | Yes |
| 62 | NM\_006372 | na | NM\_006372 Entrez,  Source | NULL | 141 | 3.319 | 0.2651 | Yes |
| 63 | Contig44289\_RC | na | CONTIG44289\_RC Entrez,  Source | NULL | 145 | 3.299 | 0.2693 | Yes |
| 64 | NM\_014750 | na | NM\_014750 Entrez,  Source | NULL | 147 | 3.292 | 0.2736 | Yes |
| 65 | NM\_003158 | na | NM\_003158 Entrez,  Source | NULL | 152 | 3.277 | 0.2778 | Yes |
| 66 | NM\_018455 | na | NM\_018455 Entrez,  Source | NULL | 153 | 3.274 | 0.2821 | Yes |
| 67 | AK000745 | na | AK000745 Entrez,  Source | NULL | 167 | 3.223 | 0.2859 | Yes |
| 68 | Contig3902\_RC | na | CONTIG3902\_RC Entrez,  Source | NULL | 168 | 3.223 | 0.2902 | Yes |
| 69 | NM\_013296 | na | NM\_013296 Entrez,  Source | NULL | 171 | 3.207 | 0.2945 | Yes |
| 70 | Contig2399\_RC | na | CONTIG2399\_RC Entrez,  Source | NULL | 177 | 3.187 | 0.2986 | Yes |
| 71 | NM\_004336 | na | NM\_004336 Entrez,  Source | NULL | 181 | 3.172 | 0.3028 | Yes |
| 72 | NM\_000788 | na | NM\_000788 Entrez,  Source | NULL | 185 | 3.162 | 0.3070 | Yes |
| 73 | NM\_001124 | na | NM\_001124 Entrez,  Source | NULL | 187 | 3.155 | 0.3113 | Yes |
| 74 | NM\_019013 | na | NM\_019013 Entrez,  Source | NULL | 193 | 3.148 | 0.3154 | Yes |
| 75 | Contig55188\_RC | na | CONTIG55188\_RC Entrez,  Source | NULL | 197 | 3.136 | 0.3196 | Yes |
| 76 | AL137718 | na | AL137718 Entrez,  Source | NULL | 198 | 3.131 | 0.3239 | Yes |
| 77 | NM\_006265 | na | NM\_006265 Entrez,  Source | NULL | 203 | 3.121 | 0.3281 | Yes |
| 78 | NM\_001827 | na | NM\_001827 Entrez,  Source | NULL | 210 | 3.099 | 0.3322 | Yes |
| 79 | NM\_014968 | na | NM\_014968 Entrez,  Source | NULL | 218 | 3.070 | 0.3362 | Yes |
| 80 | Contig51519\_RC | na | CONTIG51519\_RC Entrez,  Source | NULL | 222 | 3.063 | 0.3404 | Yes |
| 81 | Contig56457\_RC | na | CONTIG56457\_RC Entrez,  Source | NULL | 224 | 3.057 | 0.3447 | Yes |
| 82 | NM\_004358 | na | NM\_004358 Entrez,  Source | NULL | 229 | 3.043 | 0.3489 | Yes |
| 83 | NM\_018454 | na | NM\_018454 Entrez,  Source | NULL | 232 | 3.035 | 0.3531 | Yes |
| 84 | NM\_003376 | na | NM\_003376 Entrez,  Source | NULL | 233 | 3.030 | 0.3575 | Yes |
| 85 | NM\_014889 | na | NM\_014889 Entrez,  Source | NULL | 240 | 3.021 | 0.3615 | Yes |
| 86 | D25328 | na | D25328 Entrez,  Source | NULL | 242 | 3.017 | 0.3658 | Yes |
| 87 | NM\_004052 | na | NM\_004052 Entrez,  Source | NULL | 246 | 3.010 | 0.3700 | Yes |
| 88 | NM\_014078 | na | NM\_014078 Entrez,  Source | NULL | 265 | 2.962 | 0.3736 | Yes |
| 89 | NM\_018098 | na | NM\_018098 Entrez,  Source | NULL | 273 | 2.932 | 0.3777 | Yes |
| 90 | NM\_013437 | na | NM\_013437 Entrez,  Source | NULL | 276 | 2.925 | 0.3819 | Yes |
| 91 | NM\_006931 | na | NM\_006931 Entrez,  Source | NULL | 281 | 2.914 | 0.3861 | Yes |
| 92 | NM\_002073 | na | NM\_002073 Entrez,  Source | NULL | 282 | 2.914 | 0.3904 | Yes |
| 93 | NM\_003676 | na | NM\_003676 Entrez,  Source | NULL | 300 | 2.887 | 0.3940 | Yes |
| 94 | NM\_001333 | na | NM\_001333 Entrez,  Source | NULL | 303 | 2.883 | 0.3983 | Yes |
| 95 | NM\_000599 | na | NM\_000599 Entrez,  Source | NULL | 319 | 2.848 | 0.4020 | Yes |
| 96 | NM\_000436 | na | NM\_000436 Entrez,  Source | NULL | 323 | 2.835 | 0.4062 | Yes |
| 97 | NM\_004702 | na | NM\_004702 Entrez,  Source | NULL | 325 | 2.831 | 0.4105 | Yes |
| 98 | NM\_014754 | na | NM\_014754 Entrez,  Source | NULL | 328 | 2.828 | 0.4147 | Yes |
| 99 | Contig57864\_RC | na | CONTIG57864\_RC Entrez,  Source | NULL | 347 | 2.794 | 0.4183 | Yes |
| 100 | Contig17359\_RC | na | CONTIG17359\_RC Entrez,  Source | NULL | 360 | 2.786 | 0.4221 | Yes |
| 101 | Contig21812\_RC | na | CONTIG21812\_RC Entrez,  Source | NULL | 361 | 2.783 | 0.4265 | Yes |
| 102 | Contig50106\_RC | na | CONTIG50106\_RC Entrez,  Source | NULL | 372 | 2.755 | 0.4304 | Yes |
| 103 | Contig64688 | na | CONTIG64688 Entrez,  Source | NULL | 395 | 2.719 | 0.4338 | Yes |
| 104 | NM\_004603 | na | NM\_004603 Entrez,  Source | NULL | 401 | 2.706 | 0.4379 | Yes |
| 105 | AL133603 | na | AL133603 Entrez,  Source | NULL | 422 | 2.687 | 0.4414 | Yes |
| 106 | AF052159 | na | AF052159 Entrez,  Source | NULL | 423 | 2.684 | 0.4458 | Yes |
| 107 | AF155117 | na | AF155117 Entrez,  Source | NULL | 443 | 2.642 | 0.4493 | Yes |
| 108 | X94232 | na | X94232 Entrez,  Source | NULL | 453 | 2.629 | 0.4533 | Yes |
| 109 | Contig60864\_RC | na | CONTIG60864\_RC Entrez,  Source | NULL | 461 | 2.613 | 0.4573 | Yes |
| 110 | AL137502 | na | AL137502 Entrez,  Source | NULL | 464 | 2.608 | 0.4616 | Yes |
| 111 | NM\_006281 | na | NM\_006281 Entrez,  Source | NULL | 476 | 2.593 | 0.4654 | Yes |
| 112 | NM\_017779 | na | NM\_017779 Entrez,  Source | NULL | 492 | 2.565 | 0.4691 | Yes |
| 113 | NM\_018120 | na | NM\_018120 Entrez,  Source | NULL | 512 | 2.545 | 0.4727 | Yes |
| 114 | NM\_014791 | na | NM\_014791 Entrez,  Source | NULL | 532 | 2.515 | 0.4762 | Yes |
| 115 | U58033 | na | U58033 Entrez,  Source | NULL | 534 | 2.512 | 0.4805 | Yes |
| 116 | NM\_007203 | na | NM\_007203 Entrez,  Source | NULL | 538 | 2.508 | 0.4847 | Yes |
| 117 | NM\_012214 | na | NM\_012214 Entrez,  Source | NULL | 590 | 2.447 | 0.4869 | Yes |
| 118 | NM\_000096 | na | NM\_000096 Entrez,  Source | NULL | 603 | 2.433 | 0.4908 | Yes |
| 119 | AL137295 | na | AL137295 Entrez,  Source | NULL | 617 | 2.421 | 0.4946 | Yes |
| 120 | Contig55313\_RC | na | CONTIG55313\_RC Entrez,  Source | NULL | 622 | 2.416 | 0.4987 | Yes |
| 121 | NM\_002019 | na | NM\_002019 Entrez,  Source | NULL | 626 | 2.411 | 0.5029 | Yes |
| 122 | NM\_020675 | na | NM\_020675 Entrez,  Source | NULL | 640 | 2.397 | 0.5067 | Yes |
| 123 | NM\_003607 | na | NM\_003607 Entrez,  Source | NULL | 673 | 2.354 | 0.5097 | Yes |
| 124 | Contig40128\_RC | na | CONTIG40128\_RC Entrez,  Source | NULL | 714 | 2.312 | 0.5124 | Yes |
| 125 | AL080110 | na | AL080110 Entrez,  Source | NULL | 723 | 2.304 | 0.5164 | Yes |
| 126 | AL080079 | na | AL080079 Entrez,  Source | NULL | 827 | 2.207 | 0.5165 | Yes |
| 127 | Contig40831\_RC | na | CONTIG40831\_RC Entrez,  Source | NULL | 834 | 2.200 | 0.5206 | Yes |
| 128 | Contig58368\_RC | na | CONTIG58368\_RC Entrez,  Source | NULL | 839 | 2.192 | 0.5248 | Yes |
| 129 | Contig1778\_RC | na | CONTIG1778\_RC Entrez,  Source | NULL | 873 | 2.165 | 0.5277 | Yes |
| 130 | NM\_000158 | na | NM\_000158 Entrez,  Source | NULL | 888 | 2.148 | 0.5315 | Yes |
| 131 | L27560 | na | L27560 Entrez,  Source | NULL | 946 | 2.107 | 0.5335 | Yes |
| 132 | NM\_014109 | na | NM\_014109 Entrez,  Source | NULL | 966 | 2.094 | 0.5370 | Yes |
| 133 | NM\_012177 | na | NM\_012177 Entrez,  Source | NULL | 990 | 2.075 | 0.5404 | Yes |
| 134 | Contig4595 | na | CONTIG4595 Entrez,  Source | NULL | 1000 | 2.070 | 0.5443 | Yes |
| 135 | Contig50410 | na | CONTIG50410 Entrez,  Source | NULL | 1013 | 2.065 | 0.5482 | Yes |
| 136 | Contig63649\_RC | na | CONTIG63649\_RC Entrez,  Source | NULL | 1030 | 2.049 | 0.5518 | Yes |
| 137 | NM\_018004 | na | NM\_018004 Entrez,  Source | NULL | 1055 | 2.032 | 0.5552 | Yes |
| 138 | R70506\_RC | na | R70506\_RC Entrez,  Source | NULL | 1109 | 1.989 | 0.5573 | Yes |
| 139 | NM\_007036 | na | NM\_007036 Entrez,  Source | NULL | 1124 | 1.974 | 0.5611 | Yes |
| 140 | Contig13480\_RC | na | CONTIG13480\_RC Entrez,  Source | NULL | 1170 | 1.935 | 0.5635 | Yes |
| 141 | Contig44799\_RC | na | CONTIG44799\_RC Entrez,  Source | NULL | 1187 | 1.926 | 0.5672 | Yes |
| 142 | Contig2504\_RC | na | CONTIG2504\_RC Entrez,  Source | NULL | 1214 | 1.903 | 0.5705 | Yes |
| 143 | AB032973 | na | AB032973 Entrez,  Source | NULL | 1260 | 1.876 | 0.5729 | Yes |
| 144 | Contig53226\_RC | na | CONTIG53226\_RC Entrez,  Source | NULL | 1266 | 1.872 | 0.5771 | Yes |
| 145 | NM\_006096 | na | NM\_006096 Entrez,  Source | NULL | 1347 | 1.818 | 0.5781 | Yes |
| 146 | NM\_003234 | na | NM\_003234 Entrez,  Source | NULL | 1491 | 1.757 | 0.5765 | Yes |
| 147 | Contig46802\_RC | na | CONTIG46802\_RC Entrez,  Source | NULL | 1505 | 1.753 | 0.5803 | Yes |
| 148 | Contig45816\_RC | na | CONTIG45816\_RC Entrez,  Source | NULL | 1816 | 1.589 | 0.5719 | No |
| 149 | M21551 | na | M21551 Entrez,  Source | NULL | 3712 | 1.409 | 0.4981 | No |
| 150 | Contig46653\_RC | na | CONTIG46653\_RC Entrez,  Source | NULL | 4242 | 1.283 | 0.4806 | No |
| 151 | Contig8581\_RC | na | CONTIG8581\_RC Entrez,  Source | NULL | 4308 | 1.259 | 0.4822 | No |
| 152 | NM\_006681 | na | NM\_006681 Entrez,  Source | NULL | 4608 | 1.138 | 0.4742 | No |
| 153 | Contig33814\_RC | na | CONTIG33814\_RC Entrez,  Source | NULL | 4749 | 1.099 | 0.4728 | No |
| 154 | Contig20217\_RC | na | CONTIG20217\_RC Entrez,  Source | NULL | 4786 | 1.091 | 0.4756 | No |
| 155 | NM\_020386 | na | NM\_020386 Entrez,  Source | NULL | 4794 | 1.089 | 0.4797 | No |
| 156 | NM\_005496 | na | NM\_005496 Entrez,  Source | NULL | 4987 | 1.061 | 0.4761 | No |
| 157 | NM\_014363 | na | NM\_014363 Entrez,  Source | NULL | 5683 | 1.030 | 0.4517 | No |
| 158 | NM\_002900 | na | NM\_002900 Entrez,  Source | NULL | 13262 | 0.959 | 0.1436 | No |
| 159 | NM\_018401 | na | NM\_018401 Entrez,  Source | NULL | 13588 | 0.931 | 0.1345 | No |
| 160 | Contig49670\_RC | na | CONTIG49670\_RC Entrez,  Source | NULL | 22893 | -1.757 | -0.2448 | No |
| 161 | Contig45347\_RC | na | CONTIG45347\_RC Entrez,  Source | NULL | 23557 | -2.168 | -0.2679 | No |
| 162 | NM\_015416 | na | NM\_015416 Entrez,  Source | NULL | 23670 | -2.268 | -0.2681 | No |
| 163 | Contig63102\_RC | na | CONTIG63102\_RC Entrez,  Source | NULL | 23747 | -2.338 | -0.2669 | No |
| 164 | AB037745 | na | AB037745 Entrez,  Source | NULL | 23766 | -2.353 | -0.2634 | No |
| 165 | Contig55829\_RC | na | CONTIG55829\_RC Entrez,  Source | NULL | 23794 | -2.373 | -0.2601 | No |
| 166 | NM\_004480 | na | NM\_004480 Entrez,  Source | NULL | 23957 | -2.550 | -0.2625 | No |
| 167 | Contig53268\_RC | na | CONTIG53268\_RC Entrez,  Source | NULL | 24082 | -2.707 | -0.2633 | No |
| 168 | Contig41887\_RC | na | CONTIG41887\_RC Entrez,  Source | NULL | 24084 | -2.707 | -0.2590 | No |
| 169 | Contig50802\_RC | na | CONTIG50802\_RC Entrez,  Source | NULL | 24089 | -2.713 | -0.2548 | No |
| 170 | Contig42421\_RC | na | CONTIG42421\_RC Entrez,  Source | NULL | 24092 | -2.719 | -0.2506 | No |
| 171 | Contig25055\_RC | na | CONTIG25055\_RC Entrez,  Source | NULL | 24126 | -2.761 | -0.2476 | No |
| 172 | Contig37063\_RC | na | CONTIG37063\_RC Entrez,  Source | NULL | 24148 | -2.799 | -0.2442 | No |
| 173 | Contig753\_RC | na | CONTIG753\_RC Entrez,  Source | NULL | 24168 | -2.822 | -0.2406 | No |
| 174 | NM\_000224 | na | NM\_000224 Entrez,  Source | NULL | 24217 | -2.915 | -0.2383 | No |
| 175 | Contig55813\_RC | na | CONTIG55813\_RC Entrez,  Source | NULL | 24242 | -2.969 | -0.2349 | No |
| 176 | Contig37598 | na | CONTIG37598 Entrez,  Source | NULL | 24252 | -2.996 | -0.2310 | No |
| 177 | AL050090 | na | AL050090 Entrez,  Source | NULL | 24260 | -3.020 | -0.2269 | No |
| 178 | NM\_006117 | na | NM\_006117 Entrez,  Source | NULL | 24272 | -3.054 | -0.2230 | No |
| 179 | Contig53742\_RC | na | CONTIG53742\_RC Entrez,  Source | NULL | 24276 | -3.060 | -0.2188 | No |
| 180 | Contig53646\_RC | na | CONTIG53646\_RC Entrez,  Source | NULL | 24314 | -3.182 | -0.2160 | No |
| 181 | AF201951 | na | AF201951 Entrez,  Source | NULL | 24316 | -3.188 | -0.2117 | No |
| 182 | NM\_000507 | na | NM\_000507 Entrez,  Source | NULL | 24335 | -3.249 | -0.2082 | No |
| 183 | NM\_003882 | na | NM\_003882 Entrez,  Source | NULL | 24337 | -3.255 | -0.2039 | No |
| 184 | AL137514 | na | AL137514 Entrez,  Source | NULL | 24347 | -3.276 | -0.1999 | No |
| 185 | NM\_004798 | na | NM\_004798 Entrez,  Source | NULL | 24349 | -3.288 | -0.1956 | No |
| 186 | NM\_015417 | na | NM\_015417 Entrez,  Source | NULL | 24359 | -3.333 | -0.1917 | No |
| 187 | AB033043 | na | AB033043 Entrez,  Source | NULL | 24360 | -3.339 | -0.1873 | No |
| 188 | Contig48328\_RC | na | CONTIG48328\_RC Entrez,  Source | NULL | 24369 | -3.366 | -0.1833 | No |
| 189 | Contig51749\_RC | na | CONTIG51749\_RC Entrez,  Source | NULL | 24373 | -3.379 | -0.1791 | No |
| 190 | AB033007 | na | AB033007 Entrez,  Source | NULL | 24374 | -3.382 | -0.1748 | No |
| 191 | AB020689 | na | AB020689 Entrez,  Source | NULL | 24378 | -3.387 | -0.1706 | No |
| 192 | AF257175 | na | AF257175 Entrez,  Source | NULL | 24384 | -3.420 | -0.1665 | No |
| 193 | Contig43747\_RC | na | CONTIG43747\_RC Entrez,  Source | NULL | 24386 | -3.424 | -0.1622 | No |
| 194 | NM\_018104 | na | NM\_018104 Entrez,  Source | NULL | 24387 | -3.438 | -0.1579 | No |
| 195 | NM\_001007 | na | NM\_001007 Entrez,  Source | NULL | 24389 | -3.452 | -0.1536 | No |
| 196 | NM\_001282 | na | NM\_001282 Entrez,  Source | NULL | 24391 | -3.469 | -0.1493 | No |
| 197 | Contig34634\_RC | na | CONTIG34634\_RC Entrez,  Source | NULL | 24392 | -3.475 | -0.1450 | No |
| 198 | Contig51963 | na | CONTIG51963 Entrez,  Source | NULL | 24395 | -3.499 | -0.1407 | No |
| 199 | AL133619 | na | AL133619 Entrez,  Source | NULL | 24398 | -3.511 | -0.1365 | No |
| 200 | Contig27312\_RC | na | CONTIG27312\_RC Entrez,  Source | NULL | 24400 | -3.517 | -0.1322 | No |
| 201 | NM\_016337 | na | NM\_016337 Entrez,  Source | NULL | 24406 | -3.539 | -0.1281 | No |
| 202 | NM\_004911 | na | NM\_004911 Entrez,  Source | NULL | 24408 | -3.551 | -0.1238 | No |
| 203 | Contig32125\_RC | na | CONTIG32125\_RC Entrez,  Source | NULL | 24410 | -3.568 | -0.1195 | No |
| 204 | NM\_002570 | na | NM\_002570 Entrez,  Source | NULL | 24414 | -3.583 | -0.1153 | No |
| 205 | NM\_000320 | na | NM\_000320 Entrez,  Source | NULL | 24419 | -3.617 | -0.1111 | No |
| 206 | AL355708 | na | AL355708 Entrez,  Source | NULL | 24420 | -3.619 | -0.1068 | No |
| 207 | NM\_003748 | na | NM\_003748 Entrez,  Source | NULL | 24421 | -3.624 | -0.1025 | No |
| 208 | NM\_013262 | na | NM\_013262 Entrez,  Source | NULL | 24422 | -3.627 | -0.0981 | No |
| 209 | NM\_012261 | na | NM\_012261 Entrez,  Source | NULL | 24428 | -3.676 | -0.0940 | No |
| 210 | NM\_020244 | na | NM\_020244 Entrez,  Source | NULL | 24430 | -3.683 | -0.0897 | No |
| 211 | AF148505 | na | AF148505 Entrez,  Source | NULL | 24431 | -3.691 | -0.0854 | No |
| 212 | NM\_004163 | na | NM\_004163 Entrez,  Source | NULL | 24433 | -3.699 | -0.0811 | No |
| 213 | Contig57595 | na | CONTIG57595 Entrez,  Source | NULL | 24441 | -3.766 | -0.0771 | No |
| 214 | Contig47405\_RC | na | CONTIG47405\_RC Entrez,  Source | NULL | 24443 | -3.796 | -0.0728 | No |
| 215 | NM\_016569 | na | NM\_016569 Entrez,  Source | NULL | 24447 | -3.832 | -0.0686 | No |
| 216 | AJ224741 | na | AJ224741 Entrez,  Source | NULL | 24448 | -3.843 | -0.0642 | No |
| 217 | Contig46223\_RC | na | CONTIG46223\_RC Entrez,  Source | NULL | 24450 | -3.852 | -0.0599 | No |
| 218 | Contig55377\_RC | na | CONTIG55377\_RC Entrez,  Source | NULL | 24454 | -3.924 | -0.0557 | No |
| 219 | AB037863 | na | AB037863 Entrez,  Source | NULL | 24455 | -3.946 | -0.0514 | No |
| 220 | Contig44064\_RC | na | CONTIG44064\_RC Entrez,  Source | NULL | 24456 | -3.957 | -0.0471 | No |
| 221 | U82987 | na | U82987 Entrez,  Source | NULL | 24457 | -3.981 | -0.0428 | No |
| 222 | U45975 | na | U45975 Entrez,  Source | NULL | 24459 | -3.992 | -0.0385 | No |
| 223 | NM\_012429 | na | NM\_012429 Entrez,  Source | NULL | 24461 | -4.019 | -0.0342 | No |
| 224 | NM\_006763 | na | NM\_006763 Entrez,  Source | NULL | 24462 | -4.049 | -0.0298 | No |
| 225 | NM\_000849 | na | NM\_000849 Entrez,  Source | NULL | 24463 | -4.050 | -0.0255 | No |
| 226 | NM\_000017 | na | NM\_000017 Entrez,  Source | NULL | 24464 | -4.065 | -0.0212 | No |
| 227 | NM\_003239 | na | NM\_003239 Entrez,  Source | NULL | 24467 | -4.134 | -0.0169 | No |
| 228 | NM\_001280 | na | NM\_001280 Entrez,  Source | NULL | 24469 | -4.220 | -0.0127 | No |
| 229 | NM\_000286 | na | NM\_000286 Entrez,  Source | NULL | 24473 | -4.367 | -0.0085 | No |
| 230 | NM\_003862 | na | NM\_003862 Entrez,  Source | NULL | 24479 | -4.656 | -0.0043 | No |
| 231 | NM\_020974 | na | NM\_020974 Entrez,  Source | NULL | 24480 | -4.823 | 0.0000 | No |
Table: GSEA details [plain text format]

  

Fig 2: VANT VEER 231      
 Blue-Pink O' Gram in the Space of the Analyzed GeneSet

  

Fig 3: VANT VEER 231: Random ES distribution      
 Gene set null distribution of ES for **VANT VEER 231**

  
