# Supplementary material for: A voting approach to identify a small number of highly predictive genes using multiple classifiers
Source: BMC Bioinformatics. 2009 Jan 30;10(Suppl 1):S19. doi: 10.1186/1471-2105-10-S1-S19 (PMC2648737; doi:10.1186/1471-2105-10-S1-S19)
Supplement: Additional file 2 — This file contains the result of gene set enrichment analysis (GSEA). [file 1471-2105-10-S1-S19-S2.zip › VANT VEER 70.html]

Details for gene set VANT VEER 70[GSEA]

|  || Dataset | dataset.phenotype.cls #relapse\_versus\_non-relapse.phenotype.cls #relapse\_versus\_non-relapse\_repos |
| Phenotype | phenotype.cls#relapse\_versus\_non-relapse\_repos |
| Upregulated in class | 0 |
| GeneSet | VANT VEER 70 |
| Enrichment Score (ES) | -0.9266648 |
| Normalized Enrichment Score (NES) | -2.5394018 |
| Nominal p-value | 0.0 |
| FDR q-value | 0.0 |
| FWER p-Value | 0.0 |
Table: GSEA Results Summary

  

Fig 1: Enrichment plot: VANT VEER 70      
 Profile of the Running ES Score & Positions of GeneSet Members on the Rank Ordered List

  

| PROBE | DESCRIPTION (from dataset) | GENE SYMBOL | GENE\_TITLE | RANK IN GENE LIST | RANK METRIC SCORE | RUNNING ES | CORE ENRICHMENT || 1 | NM\_002900 | na | NM\_002900 Entrez,  Source | NULL | 13262 | 0.959 | -0.5290 | No |
| 2 | NM\_018401 | na | NM\_018401 Entrez,  Source | NULL | 13588 | 0.931 | -0.5280 | No |
| 3 | Contig49670\_RC | na | CONTIG49670\_RC Entrez,  Source | NULL | 22893 | -1.757 | -0.8949 | No |
| 4 | NM\_015416 | na | NM\_015416 Entrez,  Source | NULL | 23670 | -2.268 | -0.9124 | Yes |
| 5 | Contig63102\_RC | na | CONTIG63102\_RC Entrez,  Source | NULL | 23747 | -2.338 | -0.9012 | Yes |
| 6 | AB037745 | na | AB037745 Entrez,  Source | NULL | 23766 | -2.353 | -0.8877 | Yes |
| 7 | NM\_004480 | na | NM\_004480 Entrez,  Source | NULL | 23957 | -2.550 | -0.8812 | Yes |
| 8 | Contig53268\_RC | na | CONTIG53268\_RC Entrez,  Source | NULL | 24082 | -2.707 | -0.8720 | Yes |
| 9 | Contig41887\_RC | na | CONTIG41887\_RC Entrez,  Source | NULL | 24084 | -2.707 | -0.8577 | Yes |
| 10 | Contig50802\_RC | na | CONTIG50802\_RC Entrez,  Source | NULL | 24089 | -2.713 | -0.8436 | Yes |
| 11 | Contig42421\_RC | na | CONTIG42421\_RC Entrez,  Source | NULL | 24092 | -2.719 | -0.8294 | Yes |
| 12 | Contig25055\_RC | na | CONTIG25055\_RC Entrez,  Source | NULL | 24126 | -2.761 | -0.8164 | Yes |
| 13 | Contig37063\_RC | na | CONTIG37063\_RC Entrez,  Source | NULL | 24148 | -2.799 | -0.8030 | Yes |
| 14 | Contig753\_RC | na | CONTIG753\_RC Entrez,  Source | NULL | 24168 | -2.822 | -0.7895 | Yes |
| 15 | NM\_000224 | na | NM\_000224 Entrez,  Source | NULL | 24217 | -2.915 | -0.7772 | Yes |
| 16 | Contig55813\_RC | na | CONTIG55813\_RC Entrez,  Source | NULL | 24242 | -2.969 | -0.7639 | Yes |
| 17 | AL050090 | na | AL050090 Entrez,  Source | NULL | 24260 | -3.020 | -0.7503 | Yes |
| 18 | NM\_006117 | na | NM\_006117 Entrez,  Source | NULL | 24272 | -3.054 | -0.7365 | Yes |
| 19 | Contig53742\_RC | na | CONTIG53742\_RC Entrez,  Source | NULL | 24276 | -3.060 | -0.7223 | Yes |
| 20 | Contig53646\_RC | na | CONTIG53646\_RC Entrez,  Source | NULL | 24314 | -3.182 | -0.7095 | Yes |
| 21 | AF201951 | na | AF201951 Entrez,  Source | NULL | 24316 | -3.188 | -0.6953 | Yes |
| 22 | NM\_000507 | na | NM\_000507 Entrez,  Source | NULL | 24335 | -3.249 | -0.6817 | Yes |
| 23 | NM\_003882 | na | NM\_003882 Entrez,  Source | NULL | 24337 | -3.255 | -0.6675 | Yes |
| 24 | AL137514 | na | AL137514 Entrez,  Source | NULL | 24347 | -3.276 | -0.6536 | Yes |
| 25 | NM\_004798 | na | NM\_004798 Entrez,  Source | NULL | 24349 | -3.288 | -0.6393 | Yes |
| 26 | NM\_015417 | na | NM\_015417 Entrez,  Source | NULL | 24359 | -3.333 | -0.6254 | Yes |
| 27 | AB033043 | na | AB033043 Entrez,  Source | NULL | 24360 | -3.339 | -0.6111 | Yes |
| 28 | Contig48328\_RC | na | CONTIG48328\_RC Entrez,  Source | NULL | 24369 | -3.366 | -0.5972 | Yes |
| 29 | Contig51749\_RC | na | CONTIG51749\_RC Entrez,  Source | NULL | 24373 | -3.379 | -0.5830 | Yes |
| 30 | AB033007 | na | AB033007 Entrez,  Source | NULL | 24374 | -3.382 | -0.5687 | Yes |
| 31 | AB020689 | na | AB020689 Entrez,  Source | NULL | 24378 | -3.387 | -0.5546 | Yes |
| 32 | AF257175 | na | AF257175 Entrez,  Source | NULL | 24384 | -3.420 | -0.5405 | Yes |
| 33 | Contig43747\_RC | na | CONTIG43747\_RC Entrez,  Source | NULL | 24386 | -3.424 | -0.5262 | Yes |
| 34 | NM\_018104 | na | NM\_018104 Entrez,  Source | NULL | 24387 | -3.438 | -0.5120 | Yes |
| 35 | NM\_001007 | na | NM\_001007 Entrez,  Source | NULL | 24389 | -3.452 | -0.4977 | Yes |
| 36 | NM\_001282 | na | NM\_001282 Entrez,  Source | NULL | 24391 | -3.469 | -0.4835 | Yes |
| 37 | Contig34634\_RC | na | CONTIG34634\_RC Entrez,  Source | NULL | 24392 | -3.475 | -0.4692 | Yes |
| 38 | Contig51963 | na | CONTIG51963 Entrez,  Source | NULL | 24395 | -3.499 | -0.4550 | Yes |
| 39 | AL133619 | na | AL133619 Entrez,  Source | NULL | 24398 | -3.511 | -0.4408 | Yes |
| 40 | Contig27312\_RC | na | CONTIG27312\_RC Entrez,  Source | NULL | 24400 | -3.517 | -0.4265 | Yes |
| 41 | NM\_004911 | na | NM\_004911 Entrez,  Source | NULL | 24408 | -3.551 | -0.4125 | Yes |
| 42 | Contig32125\_RC | na | CONTIG32125\_RC Entrez,  Source | NULL | 24410 | -3.568 | -0.3983 | Yes |
| 43 | NM\_002570 | na | NM\_002570 Entrez,  Source | NULL | 24414 | -3.583 | -0.3841 | Yes |
| 44 | NM\_000320 | na | NM\_000320 Entrez,  Source | NULL | 24419 | -3.617 | -0.3700 | Yes |
| 45 | AL355708 | na | AL355708 Entrez,  Source | NULL | 24420 | -3.619 | -0.3557 | Yes |
| 46 | NM\_003748 | na | NM\_003748 Entrez,  Source | NULL | 24421 | -3.624 | -0.3414 | Yes |
| 47 | NM\_013262 | na | NM\_013262 Entrez,  Source | NULL | 24422 | -3.627 | -0.3271 | Yes |
| 48 | NM\_012261 | na | NM\_012261 Entrez,  Source | NULL | 24428 | -3.676 | -0.3131 | Yes |
| 49 | NM\_020244 | na | NM\_020244 Entrez,  Source | NULL | 24430 | -3.683 | -0.2988 | Yes |
| 50 | AF148505 | na | AF148505 Entrez,  Source | NULL | 24431 | -3.691 | -0.2845 | Yes |
| 51 | NM\_004163 | na | NM\_004163 Entrez,  Source | NULL | 24433 | -3.699 | -0.2703 | Yes |
| 52 | Contig57595 | na | CONTIG57595 Entrez,  Source | NULL | 24441 | -3.766 | -0.2563 | Yes |
| 53 | Contig47405\_RC | na | CONTIG47405\_RC Entrez,  Source | NULL | 24443 | -3.796 | -0.2420 | Yes |
| 54 | NM\_016569 | na | NM\_016569 Entrez,  Source | NULL | 24447 | -3.832 | -0.2279 | Yes |
| 55 | AJ224741 | na | AJ224741 Entrez,  Source | NULL | 24448 | -3.843 | -0.2136 | Yes |
| 56 | Contig46223\_RC | na | CONTIG46223\_RC Entrez,  Source | NULL | 24450 | -3.852 | -0.1993 | Yes |
| 57 | Contig55377\_RC | na | CONTIG55377\_RC Entrez,  Source | NULL | 24454 | -3.924 | -0.1852 | Yes |
| 58 | AB037863 | na | AB037863 Entrez,  Source | NULL | 24455 | -3.946 | -0.1709 | Yes |
| 59 | Contig44064\_RC | na | CONTIG44064\_RC Entrez,  Source | NULL | 24456 | -3.957 | -0.1566 | Yes |
| 60 | U82987 | na | U82987 Entrez,  Source | NULL | 24457 | -3.981 | -0.1423 | Yes |
| 61 | U45975 | na | U45975 Entrez,  Source | NULL | 24459 | -3.992 | -0.1281 | Yes |
| 62 | NM\_012429 | na | NM\_012429 Entrez,  Source | NULL | 24461 | -4.019 | -0.1138 | Yes |
| 63 | NM\_006763 | na | NM\_006763 Entrez,  Source | NULL | 24462 | -4.049 | -0.0995 | Yes |
| 64 | NM\_000849 | na | NM\_000849 Entrez,  Source | NULL | 24463 | -4.050 | -0.0853 | Yes |
| 65 | NM\_000017 | na | NM\_000017 Entrez,  Source | NULL | 24464 | -4.065 | -0.0710 | Yes |
| 66 | NM\_003239 | na | NM\_003239 Entrez,  Source | NULL | 24467 | -4.134 | -0.0568 | Yes |
| 67 | NM\_001280 | na | NM\_001280 Entrez,  Source | NULL | 24469 | -4.220 | -0.0425 | Yes |
| 68 | NM\_000286 | na | NM\_000286 Entrez,  Source | NULL | 24473 | -4.367 | -0.0284 | Yes |
| 69 | NM\_003862 | na | NM\_003862 Entrez,  Source | NULL | 24479 | -4.656 | -0.0143 | Yes |
| 70 | NM\_020974 | na | NM\_020974 Entrez,  Source | NULL | 24480 | -4.823 | -0.0000 | Yes |
Table: GSEA details [plain text format]

  

Fig 2: VANT VEER 70      
 Blue-Pink O' Gram in the Space of the Analyzed GeneSet

  

Fig 3: VANT VEER 70: Random ES distribution      
 Gene set null distribution of ES for **VANT VEER 70**

  
