# Supplementary material for: A voting approach to identify a small number of highly predictive genes using multiple classifiers
Source: BMC Bioinformatics. 2009 Jan 30;10(Suppl 1):S19. doi: 10.1186/1471-2105-10-S1-S19 (PMC2648737; doi:10.1186/1471-2105-10-S1-S19)
Supplement: Additional file 2 — This file contains the result of gene set enrichment analysis (GSEA). [file 1471-2105-10-S1-S19-S2.zip › VOTING.html]

Details for gene set VOTING[GSEA]

|  || Dataset | dataset.phenotype.cls #relapse\_versus\_non-relapse.phenotype.cls #relapse\_versus\_non-relapse\_repos |
| Phenotype | phenotype.cls#relapse\_versus\_non-relapse\_repos |
| Upregulated in class | 1 |
| GeneSet | VOTING |
| Enrichment Score (ES) | 0.79827577 |
| Normalized Enrichment Score (NES) | 2.1181545 |
| Nominal p-value | 0.0 |
| FDR q-value | 0.0051020407 |
| FWER p-Value | 0.01 |
Table: GSEA Results Summary

  

Fig 1: Enrichment plot: VOTING      
 Profile of the Running ES Score & Positions of GeneSet Members on the Rank Ordered List

  

| PROBE | DESCRIPTION (from dataset) | GENE SYMBOL | GENE\_TITLE | RANK IN GENE LIST | RANK METRIC SCORE | RUNNING ES | CORE ENRICHMENT || 1 | AL080059 | na | AL080059 Entrez,  Source | NULL | 0 | 6.260 | 0.1429 | Yes |
| 2 | NM\_001216 | na | NM\_001216 Entrez,  Source | NULL | 114 | 3.408 | 0.2811 | Yes |
| 3 | NM\_006681 | na | NM\_006681 Entrez,  Source | NULL | 4608 | 1.138 | 0.2404 | Yes |
| 4 | Contig33814\_RC | na | CONTIG33814\_RC Entrez,  Source | NULL | 4749 | 1.099 | 0.3775 | Yes |
| 5 | Contig20217\_RC | na | CONTIG20217\_RC Entrez,  Source | NULL | 4786 | 1.091 | 0.5189 | Yes |
| 6 | Contig11065\_RC | na | CONTIG11065\_RC Entrez,  Source | NULL | 4904 | 1.072 | 0.6570 | Yes |
| 7 | NM\_005219 | na | NM\_005219 Entrez,  Source | NULL | 4943 | 1.066 | 0.7983 | Yes |
Table: GSEA details [plain text format]

  

Fig 2: VOTING      
 Blue-Pink O' Gram in the Space of the Analyzed GeneSet

  

Fig 3: VOTING: Random ES distribution      
 Gene set null distribution of ES for **VOTING**

  
