# Supplementary figures and images for: A voting approach to identify a small number of highly predictive genes using multiple classifiers
Source: BMC Bioinformatics. 2009 Jan 30;10(Suppl 1):S19. doi: 10.1186/1471-2105-10-S1-S19 (PMC2648737; doi:10.1186/1471-2105-10-S1-S19)

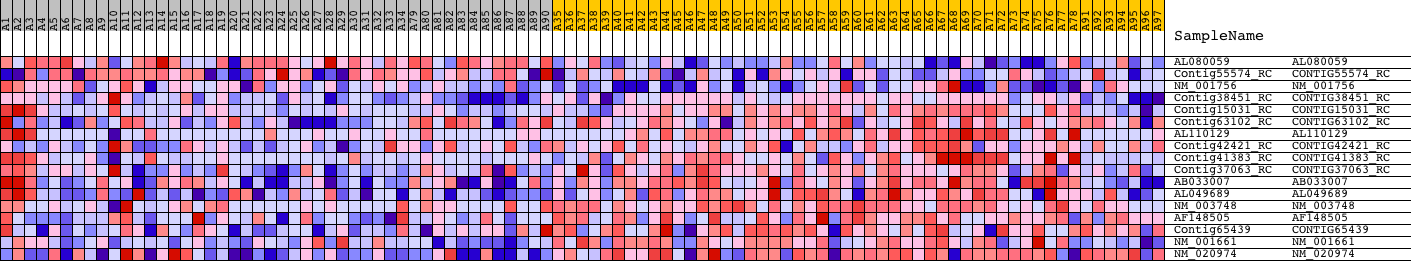

Supplement: Additional file 2 — This file contains the result of gene set enrichment analysis (GSEA). [file 1471-2105-10-S1-S19-S2.zip › ALEXE_50.png]

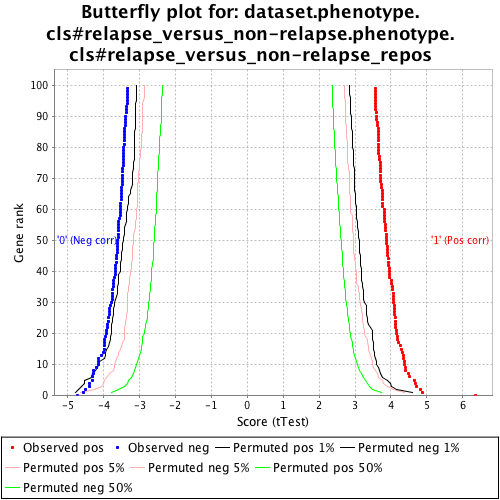

Supplement: Additional file 2 — This file contains the result of gene set enrichment analysis (GSEA). [file 1471-2105-10-S1-S19-S2.zip › butterfly_plot.png]

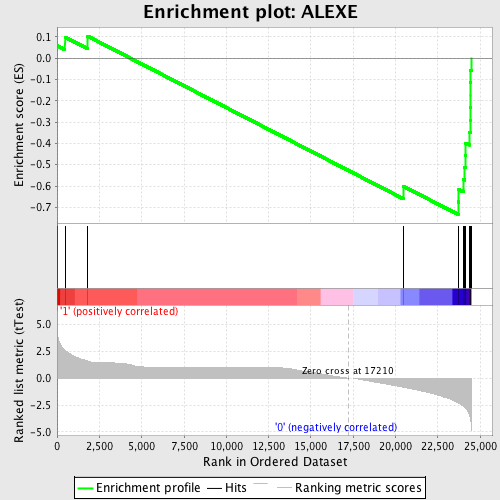

Supplement: Additional file 2 — This file contains the result of gene set enrichment analysis (GSEA). [file 1471-2105-10-S1-S19-S2.zip › enplot_ALEXE_49.png]

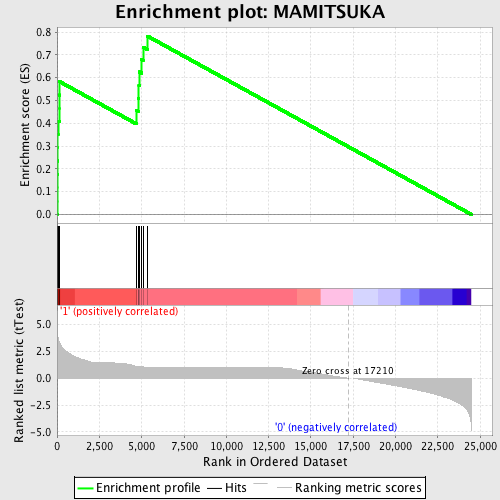

Supplement: Additional file 2 — This file contains the result of gene set enrichment analysis (GSEA). [file 1471-2105-10-S1-S19-S2.zip › enplot_MAMITSUKA_37.png]

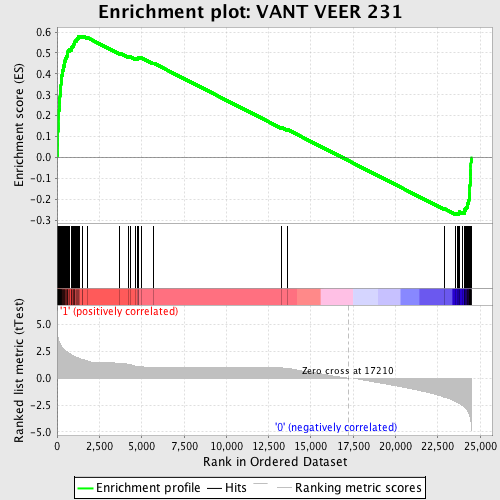

Supplement: Additional file 2 — This file contains the result of gene set enrichment analysis (GSEA). [file 1471-2105-10-S1-S19-S2.zip › enplot_VANT_VEER_231_43.png]

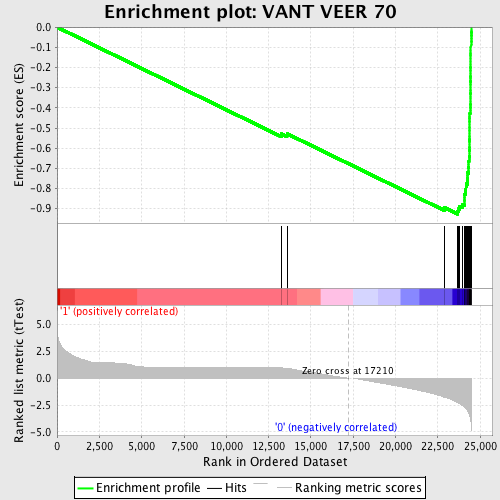

Supplement: Additional file 2 — This file contains the result of gene set enrichment analysis (GSEA). [file 1471-2105-10-S1-S19-S2.zip › enplot_VANT_VEER_70_46.png]

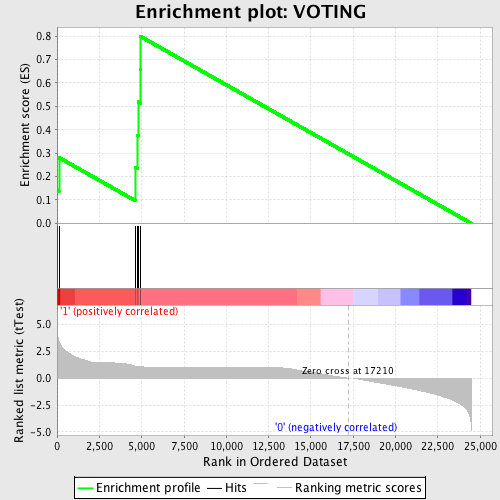

Supplement: Additional file 2 — This file contains the result of gene set enrichment analysis (GSEA). [file 1471-2105-10-S1-S19-S2.zip › enplot_VOTING_40.png]

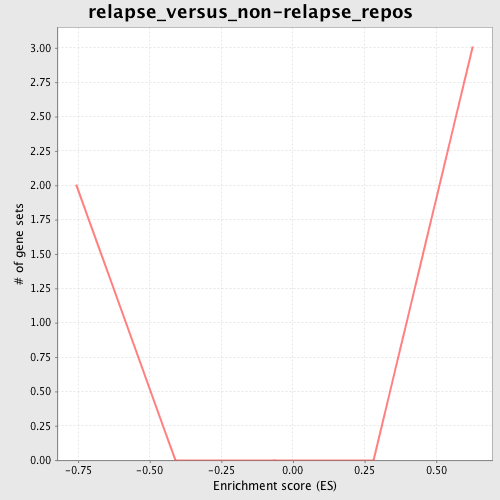

Supplement: Additional file 2 — This file contains the result of gene set enrichment analysis (GSEA). [file 1471-2105-10-S1-S19-S2.zip › global_es_histogram.png]

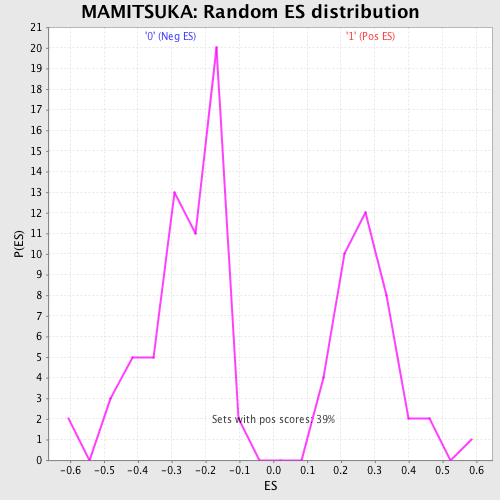

Supplement: Additional file 2 — This file contains the result of gene set enrichment analysis (GSEA). [file 1471-2105-10-S1-S19-S2.zip › gset_rnd_es_dist_39.png]

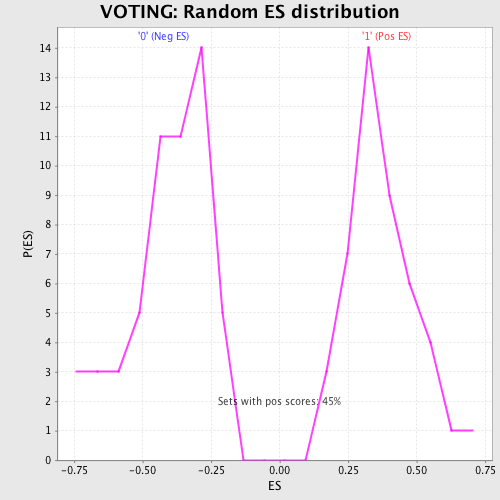

Supplement: Additional file 2 — This file contains the result of gene set enrichment analysis (GSEA). [file 1471-2105-10-S1-S19-S2.zip › gset_rnd_es_dist_42.png]

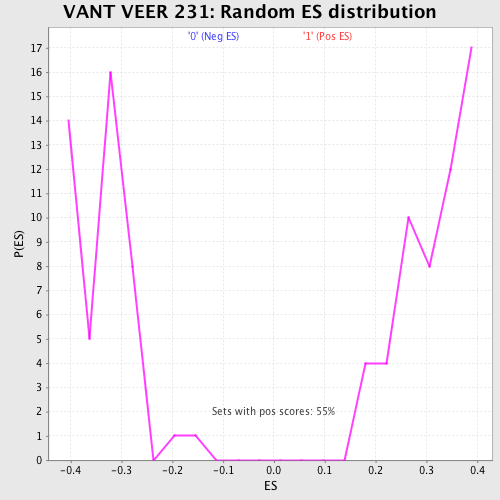

Supplement: Additional file 2 — This file contains the result of gene set enrichment analysis (GSEA). [file 1471-2105-10-S1-S19-S2.zip › gset_rnd_es_dist_45.png]

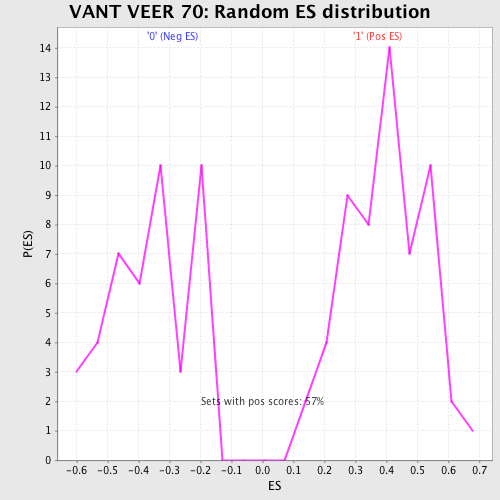

Supplement: Additional file 2 — This file contains the result of gene set enrichment analysis (GSEA). [file 1471-2105-10-S1-S19-S2.zip › gset_rnd_es_dist_48.png]

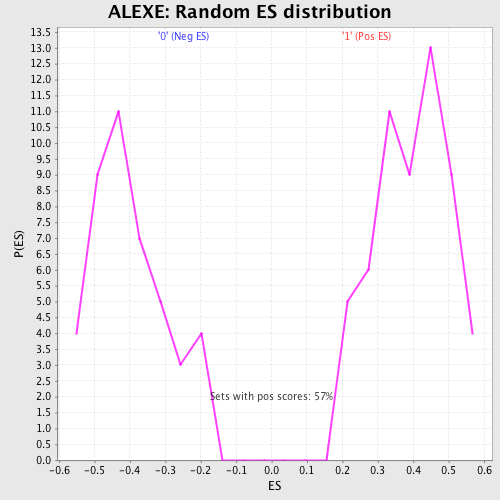

Supplement: Additional file 2 — This file contains the result of gene set enrichment analysis (GSEA). [file 1471-2105-10-S1-S19-S2.zip › gset_rnd_es_dist_51.png]

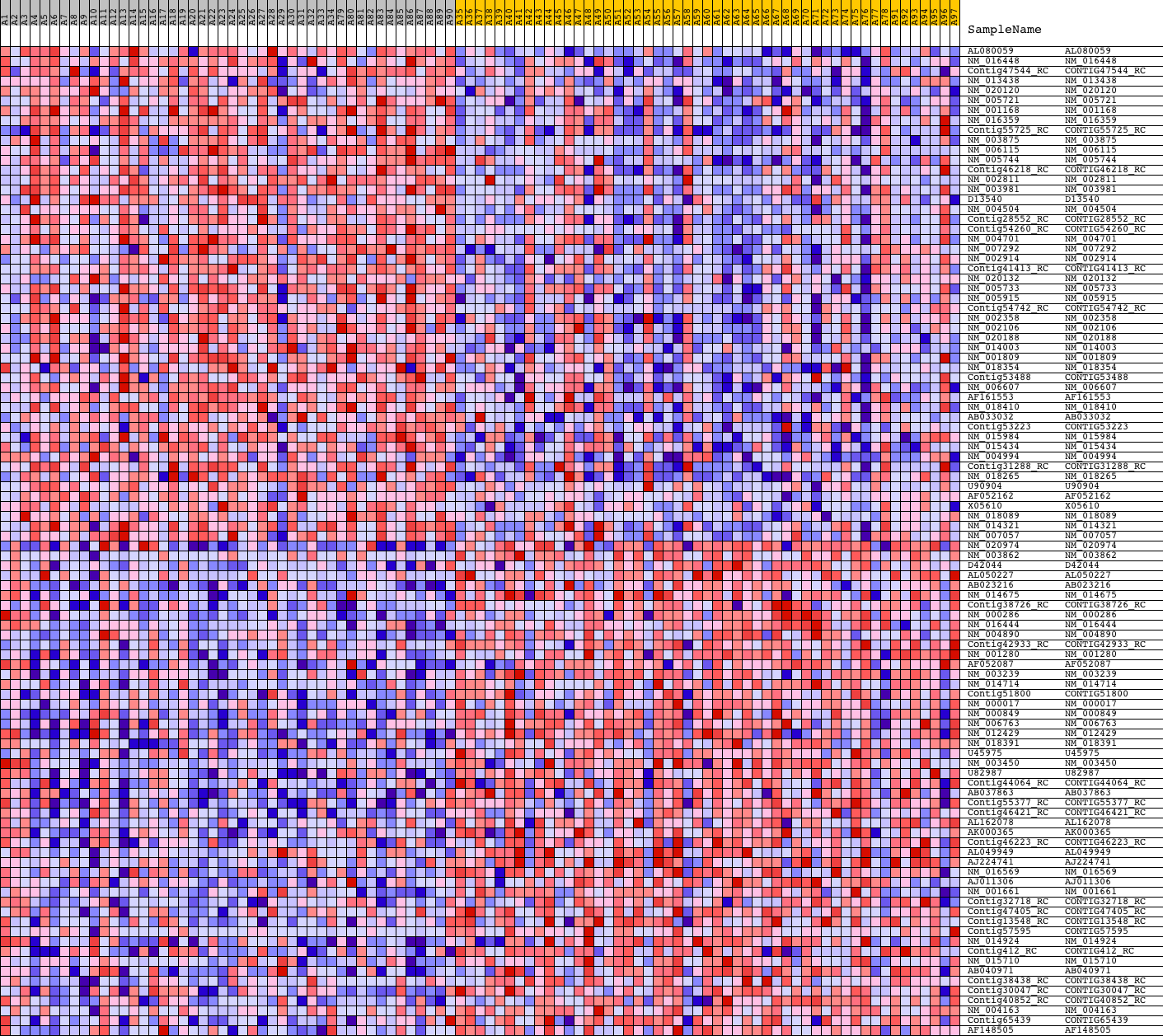

Supplement: Additional file 2 — This file contains the result of gene set enrichment analysis (GSEA). [file 1471-2105-10-S1-S19-S2.zip › heat_map_35.png]

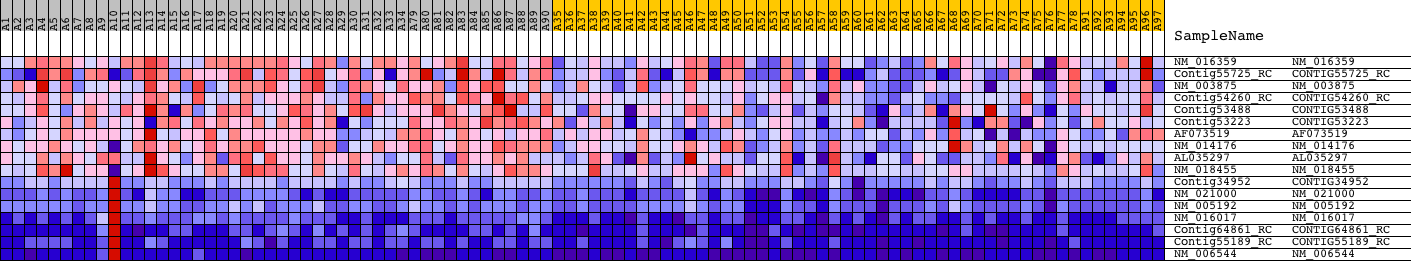

Supplement: Additional file 2 — This file contains the result of gene set enrichment analysis (GSEA). [file 1471-2105-10-S1-S19-S2.zip › MAMITSUKA_38.png]

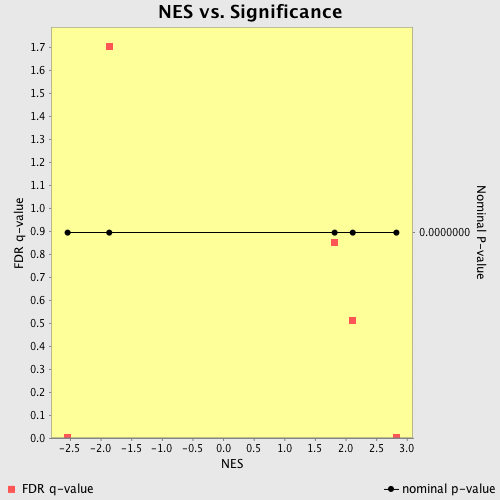

Supplement: Additional file 2 — This file contains the result of gene set enrichment analysis (GSEA). [file 1471-2105-10-S1-S19-S2.zip › pvalues_vs_nes_plot.png]

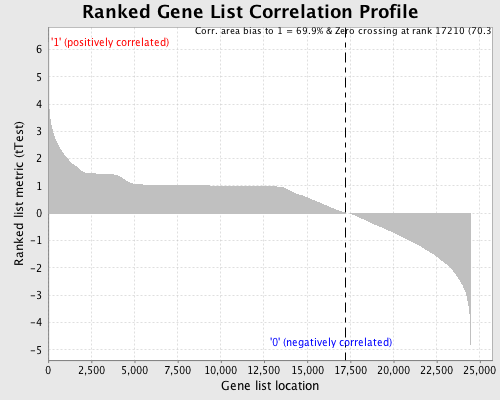

Supplement: Additional file 2 — This file contains the result of gene set enrichment analysis (GSEA). [file 1471-2105-10-S1-S19-S2.zip › ranked_list_corr_36.png]

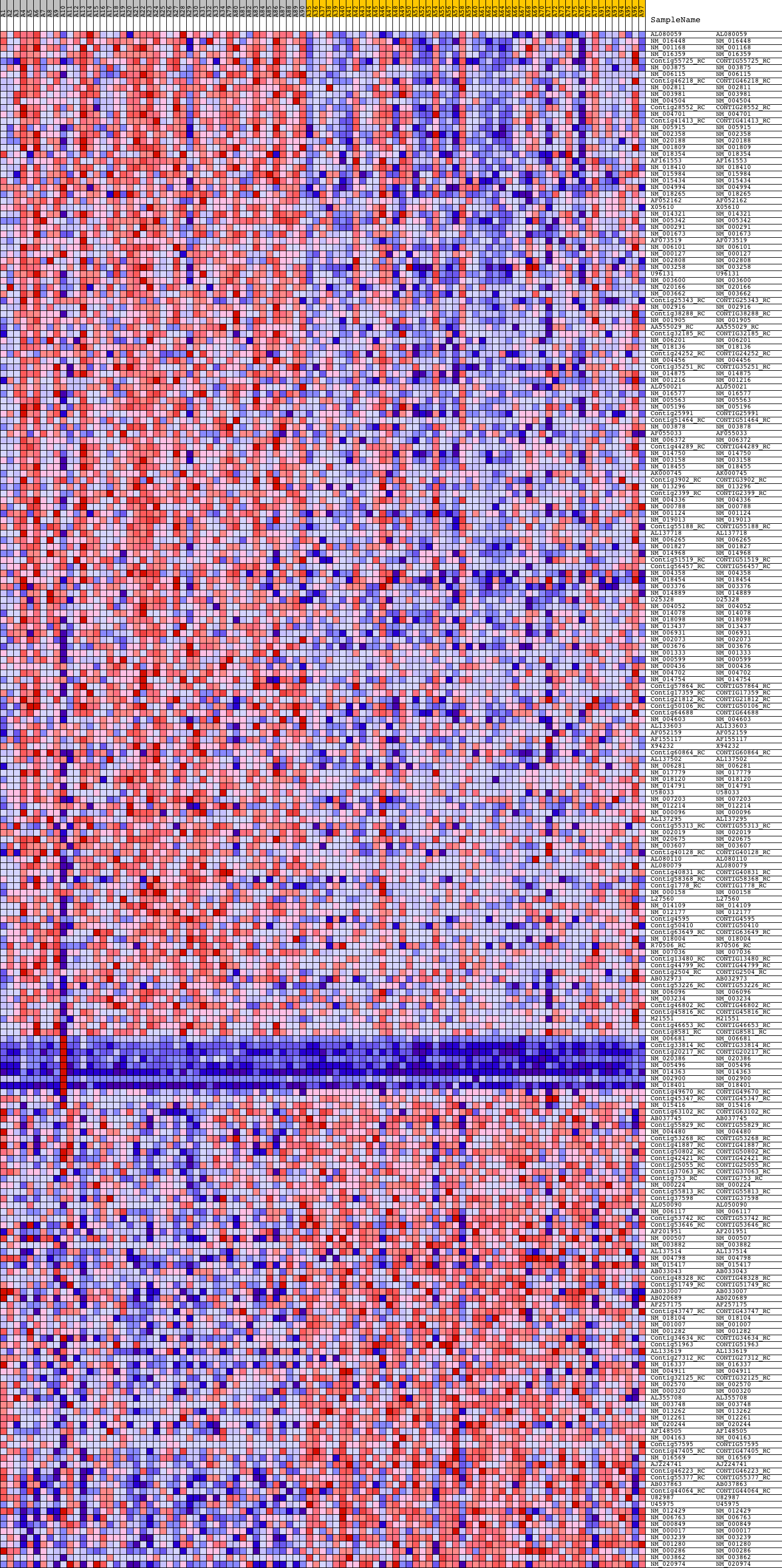

Supplement: Additional file 2 — This file contains the result of gene set enrichment analysis (GSEA). [file 1471-2105-10-S1-S19-S2.zip › VANT_VEER_231_44.png]

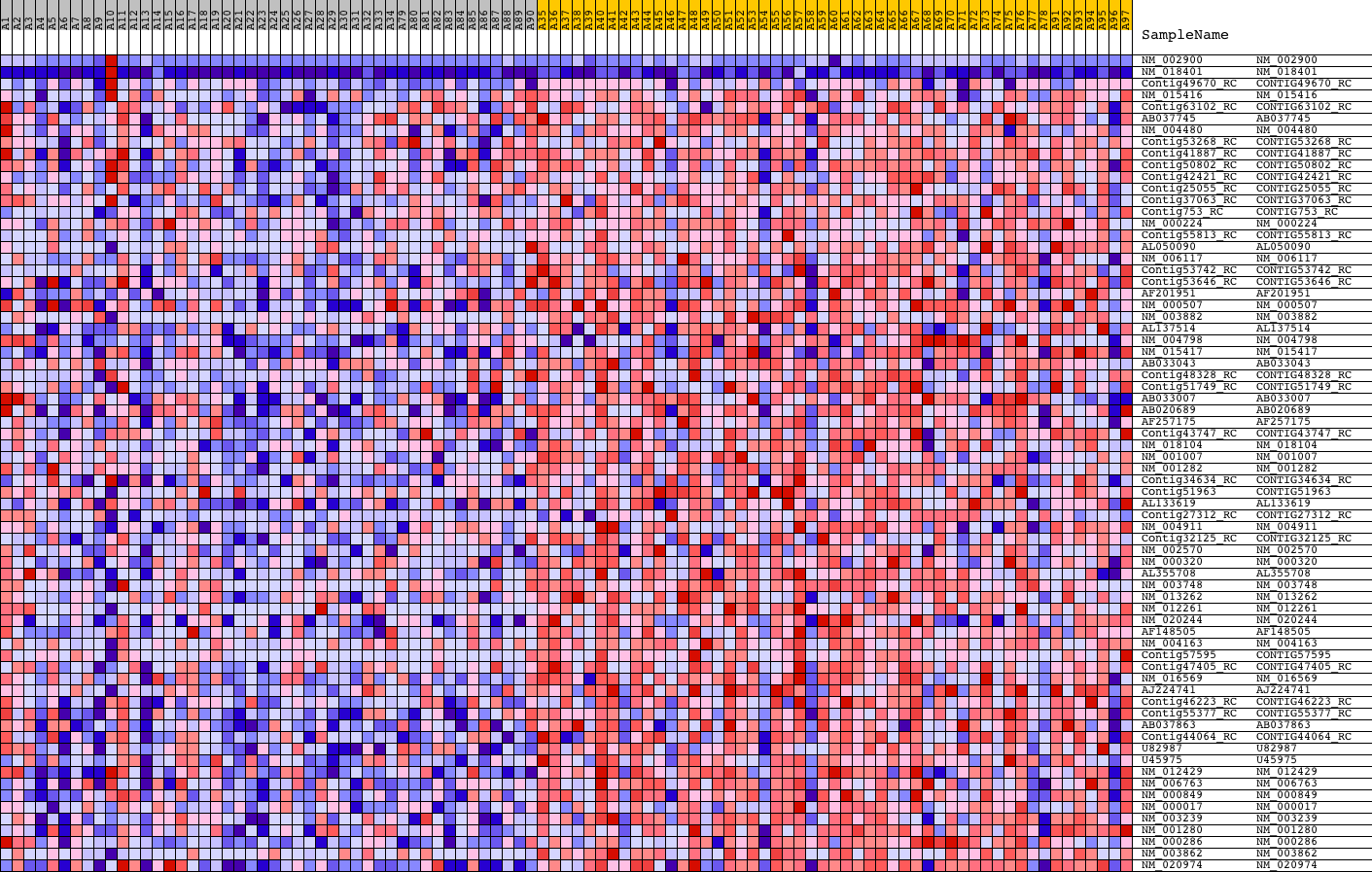

Supplement: Additional file 2 — This file contains the result of gene set enrichment analysis (GSEA). [file 1471-2105-10-S1-S19-S2.zip › VANT_VEER_70_47.png]

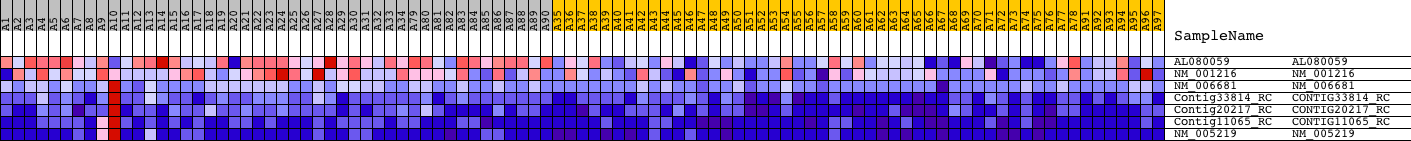

Supplement: Additional file 2 — This file contains the result of gene set enrichment analysis (GSEA). [file 1471-2105-10-S1-S19-S2.zip › VOTING_41.png]
